# BLIMP1 negatively regulates IL-2 signaling in T cells

**DOI:** 10.1126/sciadv.adx8105

**Published:** 2025-07-18

**Authors:** Suyasha Roy, Min Ren, Peng Li, Kairong Cui, Yaqiang Cao, Bryan Fisk, Tovah E. Markowitz, Neelam Redekar, Keiko Sakamoto, Keisuke Nagao, Jangsuk Oh, Rosanne Spolski, Wei Liao, Sigrid P. Dubois, Brian L. Kelsall, Keji Zhao, James D. Phelan, Warren J. Leonard

**Affiliations:** ^1^ Laboratory of Molecular Immunology and the Immunology Center, National Heart, Lung and Blood Institute, National Institutes of Health, Bethesda, MD 20892, USA.; ^2^ Laboratory of Epigenome Biology, Systems Biology Center, National Heart, Lung and Blood Institute, National Institutes of Health, Bethesda, MD 20892, USA.; ^3^ Integrated Data Sciences Section, Research Technologies Branch, National Institute of Allergy and Infectious Diseases, National Institutes of Health, Bethesda, MD 20892, USA.; ^4^ Cutaneous Leukocyte Biology Section, Dermatology Branch, National Institute of Arthritis and Musculoskeletal and Skin Diseases, National Institutes of Health, Bethesda, MD 20892, USA.; ^5^ Lymphoid Malignancies Branch, Center for Cancer Research, National Cancer Institute, National Institutes of Health, Bethesda, MD 20892, USA.; ^6^ Mucosal Immunobiology Section, Laboratory of Molecular Immunology, National Institute of Allergy and Infectious Diseases, National Institutes of Health, Bethesda, MD 20892, USA.

## Abstract

Interleukin-2 (IL-2) regulates immune homeostasis by fine-tuning the balance between effector and regulatory T (T_reg_) cells. To identify regulators of IL-2 signaling, we performed genome-wide CRISPR-knockout screening in IL-2–dependent cells derived from a patient with adult T cell leukemia (ATL) and found enrichment of single guide RNAs targeting *PRDM1*, which encodes B lymphocyte–induced maturation protein 1 (BLIMP1). BLIMP1 inhibits IL-2 production by T cells; however, its role in IL-2 signaling remains unknown. Here, we show that overexpressing *Prdm1* down-regulated IL-2 signaling, whereas *Prdm1*-deficiency enhanced IL-2 signaling in mouse CD4^+^ T cells and T_reg_ cells with augmented IL-2 signaling in T cells from influenza-infected mice and during adoptive T cell transfer–induced colitis. Deleting *PRDM1* in human CD4^+^ T cells and T_reg_ cells also increased IL-2 signaling. Furthermore, CD4^+^ T cells from patients with ATL expressed less BLIMP1 and had enhanced IL-2 signaling, whereas overexpressing *PRDM1* in ATL cells suppressed IL-2 signaling. Thus, BLIMP1 inhibits IL-2 signaling during normal and pathophysiological responses, suggesting that manipulating BLIMP1 could have therapeutic potential.

## Teaser

BLIMP1 is a negative regulator of IL-2 signaling in mouse and human T cells.

## INTRODUCTION

Interleukin-2 (IL-2) is a pleiotropic cytokine produced by antigen-activated T cells that play an important role in immune regulation, having effects on both effector (T_EFF_) and regulatory T (T_reg_) cells (*1*–*5*). IL-2 is a T cell growth factor that binds to its high-affinity IL-2 receptor (IL-2R) comprising three subunits, IL-2Rα (CD25), IL-2Rβ (CD122), and the common cytokine receptor γ-chain, IL-2Rγ (CD132) (*6*–*12*). IL-2 signaling results in the activation of signal transducers and activators of transcription 5 (STAT5), phosphatidylinositol 3-kinase (PI3K)–AKT, and mitogen-activated protein kinase–extracellular signal–regulated kinase 1/2 (ERK1/2) signaling pathways (*10*, *13*, *14*), which in turn leads to the up-regulation of genes involved in the cell survival and proliferation (*15*–*17*). IL-2 signaling also regulates immune tolerance, with *Il2*^−/−^, *Il2ra^−/−^*, and *Il2rb^−/−^* mice developing autoimmunity, while adoptive transfer of T_reg_ cells into *Il2rb^−/−^* mice prevents autoimmunity, consistent with the nonredundant role of IL-2 in the development of T_reg_ cells (*18*–*23*). IL-2 signaling initiates a negative feedback loop to limit IL-2 production and IL-2–dependent responses. The repression of *Il2* transcription is achieved in part by IL-2–dependent induction of B lymphocyte–induced maturation protein 1 (BLIMP1) and T cell receptor (TCR)–induced T-bet in T_EFF_ cells (*24*, *25*).

BLIMP1 is a transcription factor that is encoded by the gene *Prdm1* (positive regulatory domain zinc finger protein 1), originally found in B cells (*26*). BLIMP1 is induced by TCR activation and IL-2–dependent signaling in T cells, while naive T cells have lower levels of *Prdm1* mRNA expression (*27*–*29*). BLIMP1 inhibits *Il2* transcription and IL-2 production and thus participates in a negative autoregulatory feedback loop that is critical for controlling IL-2–dependent responses, particularly in situations in which it is triggered by continuous antigen exposure that results in expansion of antigen-reactive T cells (*30*–*33*). BLIMP1 is highly expressed in chronically activated T cells, which do not produce IL-2 (*34*). BLIMP1 inhibits IL-2 production not only by directly repressing *Il2* transcription but also by repressing transcription of *Fos*, which is a positive regulator of *Il2* transcription (*24*, *28*, *35*, *36*). However, whether BLIMP1 additionally regulates IL-2 signaling in T cells has not been reported.

To identify the genes whose expression might positively or negatively regulate IL-2 signaling, we performed a genome-wide CRISPR knockout screening using an IL-2–responsive cell line, ED40515(+), derived from a patient with adult T cell leukemia (ATL). ATL is an aggressive form of leukemia that develops in approximately 5% of individuals infected with human T cell lymphotropic virus-1 (HTLV-1) (*37*). In the initial phase of ATL, leukemic CD4^+^ T cells grow in an autocrine manner with persistent IL-2 expression and functional IL-2Rs (*38*). The continuous proliferation of ATL cells during this phase can be inhibited using anti-Tac monoclonal antibody directed against IL-2Rα (*39*). In the later phase, IL-2 production is lost; however, IL-2Rα expression persists, and the cells are no longer dependent on IL-2, in part due to a constitutively activated Janus kinase (JAK)–STAT signaling leading to IL-2–independent growth (*40*). In our CRISPR knockout screen, *PRDM1* (encoding BLIMP1) and *PTEN* were putatively identified as the two most significant negative regulators of IL-2–mediated cell proliferation. PTEN is a known negative regulator of IL-2R signaling (*41*, *42*), whereas BLIMP1 has not been shown to have such activity. Here, we describe the role of BLIMP1 as a negative regulator of IL-2 signaling in mouse and human T cells and in influenza infection and colitis mouse models, as well as in primary cells from patients with ATL.

## RESULTS

### CRISPR screening implicated BLIMP1 as a negative regulator of IL-2 signaling

Considering the pivotal role of IL-2 signaling in sustaining immune homeostasis and balancing T_EFF_ and T_reg_ cell responses, it is important to understand how this signaling pathway is controlled. We therefore performed a genome-wide CRISPR knockout screen using IL-2–dependent ED40515(+) cells, which are CD4^+^CD25^+^ T_reg_–like cells derived from a patient with ATL, into which Cas9 was stably expressed. The CRISPR screen (fig. S1A) was designed to identify the genes that are important for IL-2–mediated cell proliferation and survival by comparative sequence analysis of single guide RNAs (sgRNAs) in control cells versus IL-2 expanded cells. We found a significant dropout of sgRNAs for genes known to be important for IL-2 signaling, including *IL2RA*, *IL2RB*, *JAK3*, and *STAT5B*, validating the screen. sgRNAs corresponding to *PRDM1* were highly enriched, followed by those for *PTEN* (fig. S1B), making them the top hits and indicating that the deletion of these genes promoted IL-2–induced survival and proliferation of ED40515(+) cells. PTEN is known to inhibit IL-2R signaling in T_reg_ cells (*41*); however, the role of *PRDM1* (BLIMP1) in the regulation of IL-2 signaling remains unknown.

### BLIMP1 inhibits IL-2 signaling in mouse CD4^+^ T cells

**Fig. 1.**
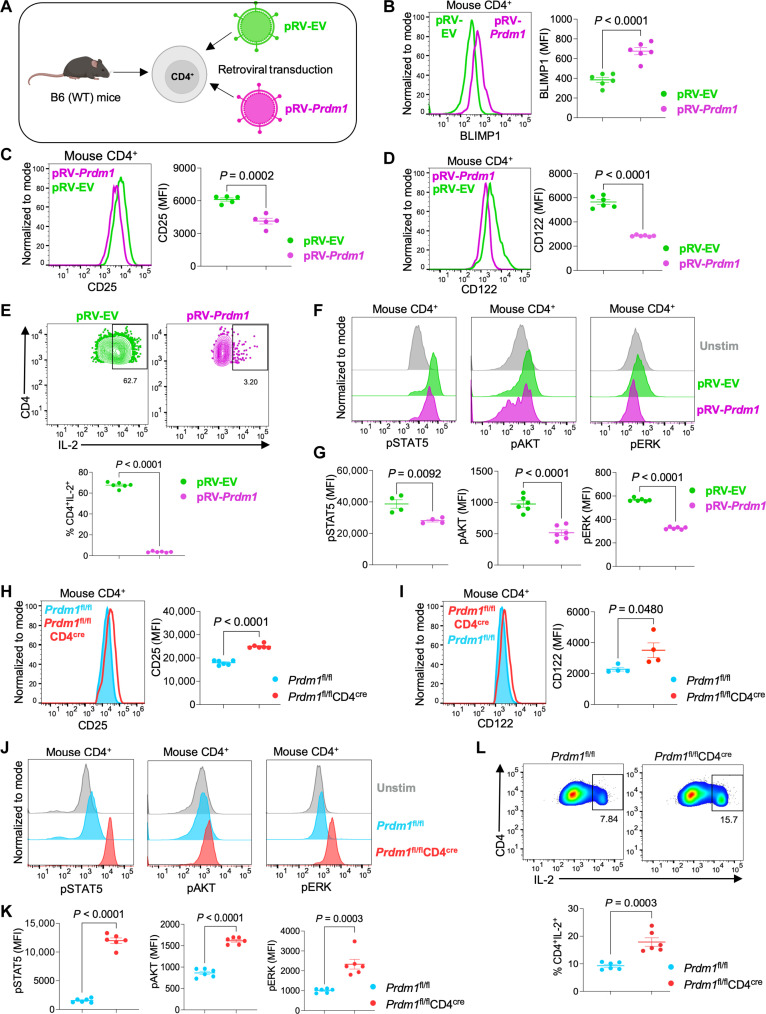
BLIMP1 inhibits IL-2 signaling in mouse CD4^+^ T cells. (**A** to **G**) CD4^+^ T cells were isolated from the spleens of WT C57BL/6 mice and TCR-stimulated for 24 hours at 37°C. Cells were retrovirally transduced with either pRV-EV or pRV-*Prdm1* and cultured with IL-2 (200 IU/ml) for 72 hours at 37°C. Cells were harvested and stained for flow cytometry analysis. (A) Experimental setup for (B) to (G), *n* = 5 to 6 individual mice per group. Created in BioRender. Roy, S. (2025) https://BioRender.com/922i5p2. [(B) to (D)] Histograms with statistical representation for (B) BLIMP1, (C) CD25, and (D) CD122 are shown. (E) Representative flow plot and statistics showing the frequency of IL-2–producing CD4^+^ T cells. (F) Histograms showing pSTAT5, pAKT, and pERK staining by flow cytometry. (G) Statistical representation of flow cytometry analysis of pSTAT5, pAKT, and pERK. Representative data (means ± SEM) from three independent experiments (*n* = 4 to 6 individual mice per group) are shown. Two-tailed unpaired Student’s *t* test was used for statistical analysis. (**H** to **L**) CD4^+^ T cells were purified from spleens of *Prdm1*^fl/fl^ and *Prdm1*^fl/fl^CD4^cre^ mice. Cells were TCR-stimulated in the presence of IL-2 (200 IU/ml) for 72 hours at 37°C, harvested, stained, and subjected to flow cytometric analysis. Shown are histograms and statistical analysis of protein expression for (H) CD25, (I) CD122, and [(J) and (K)] pSTAT5, pAKT, and pERK. (L) Representative flow plot and statistics showing the frequency of IL-2–producing CD4^+^ T cells. Data are representative of means ± SEM from three independent experiments (*n* = 4 to 6 mice per group). A two-tailed unpaired Student’s *t* test was used for statistical analysis.

To investigate the potential role of BLIMP1 in IL-2 signaling, *Prdm1* was overexpressed in mouse CD4^+^ T cells. Purified CD4^+^ T cells from the spleens of wild-type (WT) C57BL/6 mice were preactivated for 48 hours and then retrovirally transduced with pRV-EV (empty vector control) or pRV-*Prdm1 (Prdm1*–expressing vector) (Fig. 1A). There was a significant increase in BLIMP1 protein expression after retroviral transduction–mediated overexpression of *Prdm1* in mouse CD4^+^ T cells (Fig. 1B), with lower expression levels of CD25 (Fig. 1C) and CD122 (Fig. 1D). There was markedly reduced IL-2 production (Fig. 1E) and decreased phosphorylation of STAT5, AKT, and ERK (Fig. 1, F and G) after *Prdm1* overexpression in mouse CD4^+^ T cells. Thus, overexpressing BLIMP1 represses the expression of key receptor subunits and phosphorylated proteins involved in IL-2 signaling in mouse CD4^+^ T cells.

Conversely, we next eliminated *Prdm1* (BLIMP1) expression in mouse CD4^+^ T cells using *Prdm1*^fl/fl^CD4^cre^ conditional knockout (CKO) mice. CD4^+^ T cells from *Prdm1*^fl/fl^ (WT) and *Prdm1*^fl/fl^CD4^cre^ (*Prdm1*-CKO) mice were preactivated with anti-CD3 + anti-CD28 in the presence of IL-2 for 72 hours at 37°C. Although there was only a modest increase in the levels of CD25 (Fig. 1H) and CD122 (Fig. 1I) protein, the phosphorylation of STAT5, AKT, and ERK was significantly enhanced in CD4^+^ T cells from *Prdm1*-CKO mice as compared to WT mice (Fig. 1, J and K). As expected, IL-2 production was also higher after deleting *Prdm1* in mouse CD4^+^ T cells (Fig. 1L). Together, these results indicate that BLIMP1 negatively regulates IL-2 signaling in mouse CD4^+^ T cells, potentially in part by inhibiting the expression of CD25 and CD122.

### *Prdm1* knockout up-regulates IL-2 signaling in influenza virus–infected T cells

**Fig. 2.**
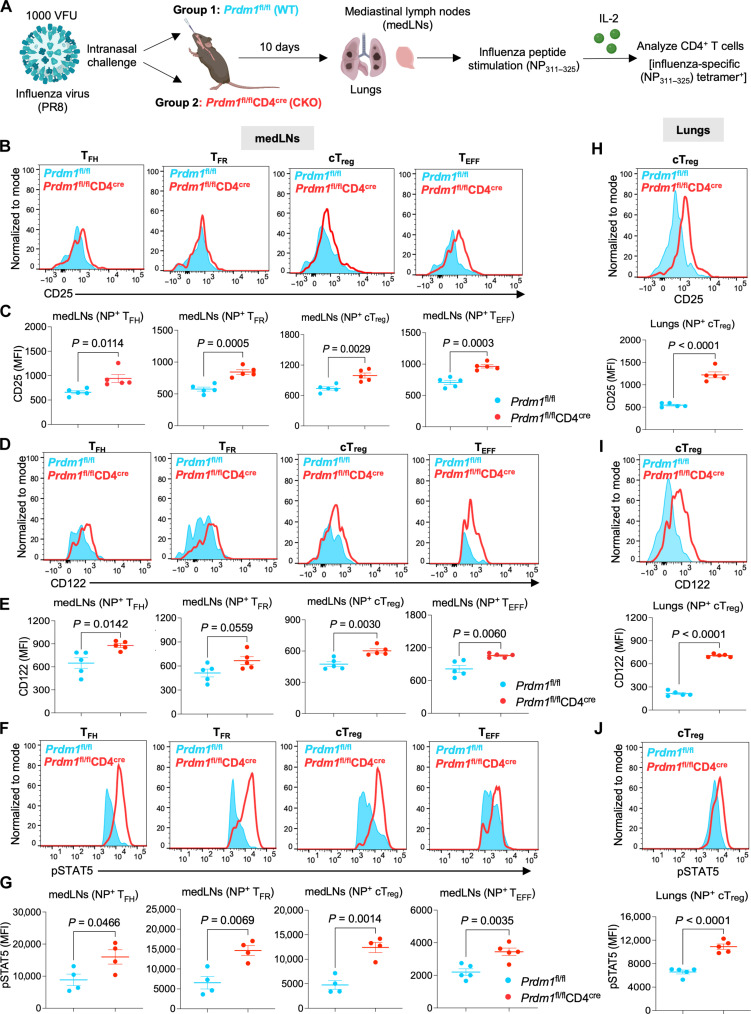
*Prdm1* knockout up-regulates IL-2 signaling in CD4^+^ T cells during influenza infection. (**A** to **J**) *Prdm1*^fl/fl^ (WT) and *Prdm1*^fl/fl^CD4^cre^ (*Prdm1*-CKO) mice were infected intranasally with 1000 viral focal units (VFU) of PR8 influenza virus. At day 10, medLNs and lungs were harvested, and single-cell suspensions were prepared. Cells were stimulated with NP_311–325_ peptide (1 μM) for 5 hours at 37°C, stained, and subjected to flow cytometry analysis. (A) Experimental setup for (B) to (J). Created in BioRender. S. Roy (2025) https://BioRender.com/ja4wpce. Flow cytometry plots with statistical representation showing [(B) and (C)] CD25, [(D) and (E)] CD122, and [(F) and (G)] pSTAT5 staining in NP_311–325_ tetramer^+^ influenza-specific follicular T helper cells (T_FH_)_,_ follicular T_reg_ cells (T_FR_)_,_ conventional T_reg_ cells (cT_reg_), and T_EFF_ cells from the medLNs of WT and *Prdm1*-CKO mice. [(H) to (J)] Histograms with statistical representation showing (H) CD25, (I) CD122, and (J) pSTAT5 staining in NP_311–325_ tetramer^+^ influenza-specific cT_reg_ cells from the lungs of WT and *Prdm1*-CKO mice by flow cytometry. Data are representative of means ± SEM from two independent experiments (*n* = 4 to 5 mice per group) are shown. Two-tailed unpaired Student’s *t* test was used for statistical analysis.

BLIMP1 is known to be essential for CD8^+^ T cell differentiation and cytotoxic function during influenza infection (*43*); however, the effect of BLIMP1 deficiency on CD4^+^ T cell responses in influenza infection has not been reported. In addition, IL-2 signaling has been shown to be induced during influenza infection (*44*). We therefore studied the role of BLIMP1 in regulating IL-2 signaling in CD4^+^ T cells from influenza-infected mice. We intranasally infected *Prdm1*^fl/fl^ (WT) and *Prdm1*^fl/fl^CD4^cre^ (CKO) mice with influenza virus strain PR8 (a pathogenic mouse-adapted strain of H1N1 influenza) and harvested mediastinal lymph nodes (medLNs) and lungs 10 days later (Fig. 2A). CD4^+^ T cells were then stimulated with IL-2 and influenza-specific peptide (nucleoprotein NP_311–325_), and NP_311–325_ tetramer–positive CD4^+^ T cell populations including T follicular helper (T_FH_), T follicular regulatory (T_FR_), T_EFF_, and conventional T_reg_ (cT_reg_) cells were analyzed (Fig. 2A). There was an increased frequency of influenza-specific total CD4^+^ T cells in both the medLNs and lungs of *Prdm1*-CKO mice as compared to WT mice (fig. S2, A to D). Among the different CD4^+^ T cell populations (gating strategy shown in fig. S3, A to C), the frequency of influenza-specific T_EFF_ cells was higher in medLNs of *Prdm1*-CKO mice as compared to WT mice (fig. S3D). The percentage of total T_FH_ and T_FR_ cell populations in the medLNs were similar in influenza-infected WT and *Prdm1*-CKO mice (fig. S3, E and F), whereas the frequency of T_reg_ cells trended slightly higher in influenza-infected *Prdm1*-infected mice but the increase was not statistically significant (fig. S3G). Nevertheless, we found higher expression of CD25 (Fig 2, B and C), CD122 (Fig. 2, D and E), and phospho-STAT5 (pSTAT5) (Fig. 2, F and G) in all populations (T_FH_, T_FR_, cT_reg_, and T_EFF_ cells) in the medLNs of influenza-infected *Prdm1*-CKO mice as compared to WT mice. There was also increased frequency of T_EFF_ and cT_reg_ cells from the lungs of influenza-infected *Prdm1*-CKO mice (fig. S3, H and I). Strikingly, there was higher expression of CD25, CD122, and pSTAT5 in cT_reg_ cells from the lungs of influenza-infected *Prdm1*-CKO mice than in WT mice (Fig. 2, H to J), as well as higher production of interferon-γ (IFN-γ) and IL-2 but lower production of IL-10 by *Prdm1*-CKO cT_reg_ cells (fig. S3, J to L), consistent with a dysregulated immune response to influenza infection. Overall, these results are consistent with BLIMP1 controlling influenza infection, at least in part, by regulating IL-2 signaling in CD4^+^ T cells.

### BLIMP1-deficient T cells have enhanced IL-2–STAT5–dependent gene expression

**Fig. 3.**
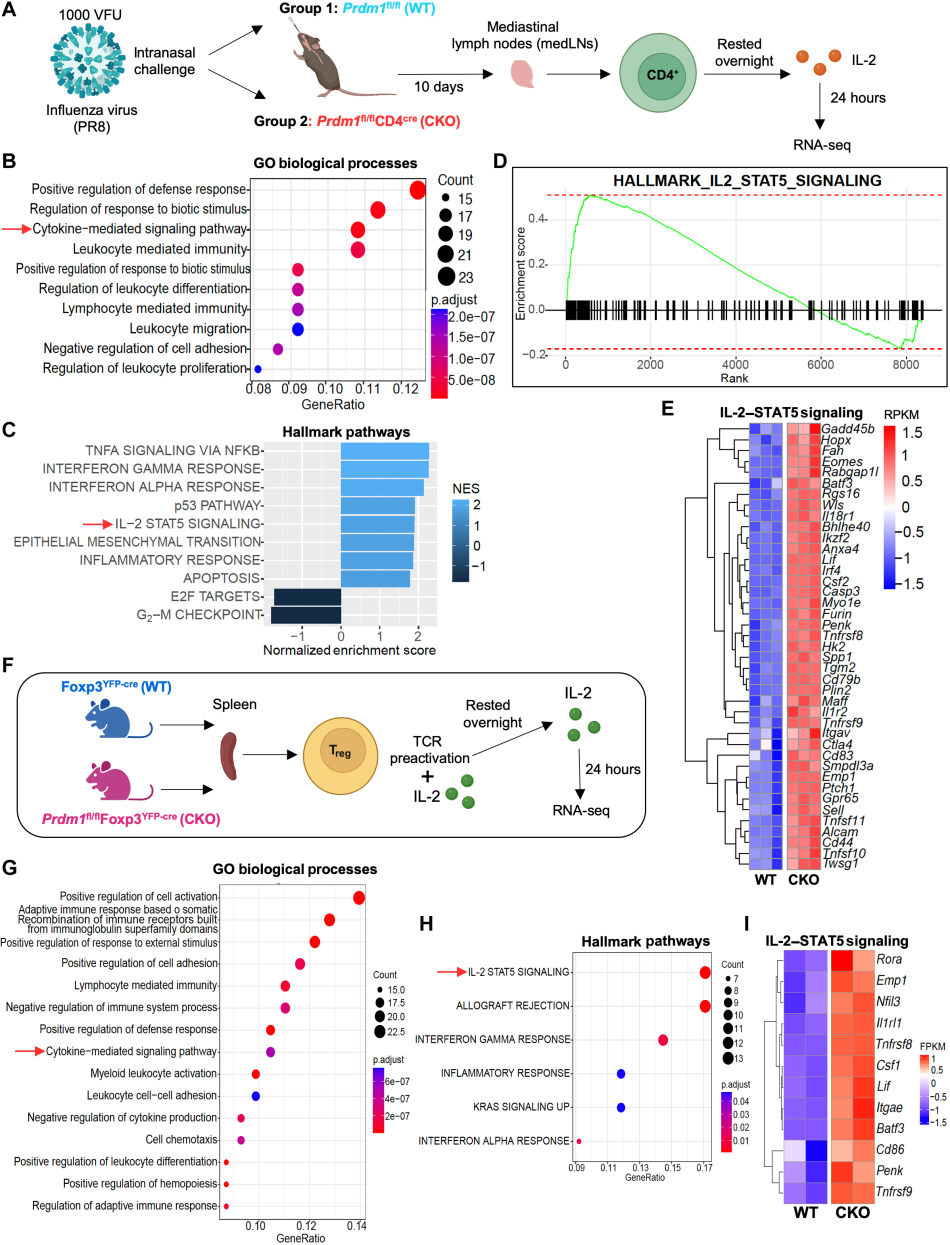
Enhanced IL-2–STAT5–dependent gene expression in BLIMP1-deficient T cells. (**A** to **E**) Mouse CD4^+^ T cells were isolated from the medLNs of influenza-infected *Prdm1*^fl/fl^ (WT) and *Prdm1*^fl/fl^CD4^cre^ (*Prdm1*-CKO) mice. Cells were stimulated ex vivo with influenza-specific peptide NP_311–325_ (1 μM), rested overnight, and stimulated with 200 IU of IL-2 for 24 hours. Cells were lysed, RNA was extracted, and RNA-seq was performed. (A) Schematic experimental design for (B) to (E). Created in BioRender. S. Roy (2025) https://BioRender.com/ydnx0k6. (B) GO based pathway over-enrichment analysis showing the differentially regulated pathways in influenza-infected CD4^+^ T cells from *Prdm1*-CKO mice and WT mice. (C) Preranked gene set enrichment analysis (GSEA) showing hallmark pathways that were up-regulated or down-regulated in influenza-infected CD4^+^ T cells from *Prdm1*-CKO mice relative to WT mice. (D) Hallmark enrichment score of IL-2–STAT5 signaling pathway in influenza-infected CD4^+^ T cells from *Prdm1*-CKO mice. (E) Heatmap showing top DEGs in the IL-2–STAT5 signaling pathway in influenza-infected CD4^+^ T cells from WT and *Prdm1*-CKO mice. (**F** to **I**) CD4^+^FOXP3^+^ T_reg_ cells were sorted from Foxp3^YFP-cre^ (WT) and *Prdm1*^fl/fl^Foxp3^YFP-cre^ (*Prdm1*-CKO) mice based on YFP expression. Cells were TCR-stimulated for 72 hours, rested overnight, stimulated with IL-2 (500 IU/ml) for 24 hours, and then lysed for RNA extraction followed by library preparation for RNA-Sequencing. (F) Schematic experimental design for (G) to (I). Created in BioRender. S. Roy (2025) https://BioRender.com/ydnx0k6. (G) GO-based over-enrichment analysis showing the differentially regulated pathways in T_reg_ cells from *Prdm1*-CKO mice as compared to WT mice. (H) Over-enrichment analysis showing differentially regulated hallmark pathways in T_reg_ cells from *Prdm1*-CKO mice relative to WT mice. (I) Heatmap showing top DEGs of the IL-2–STAT5 signaling pathway in T_reg_ cells from WT and *Prdm1*-CKO mice.

We next sought to better understand the effects of *Prdm1* deletion on IL-2–induced gene expression following influenza infection. Purified CD4^+^ T cells from medLNs of influenza-infected WT (*Prdm1*^fl/fl^) and *Prdm1*-CKO (*Prdm1*^fl/fl^CD4^cre^) mice were treated with IL-2 for 24 hours after ex vivo influenza-specific peptide (NP_311–325_) stimulation and subjected to RNA sequencing (RNA-seq) analysis (Fig. 3A). Gene ontology (GO) and hallmark pathway analysis revealed a differential regulation of cytokine-mediated signaling pathway in influenza-infected CD4^+^ T cells between *Prdm1*-CKO and WT mice (Fig. 3B), with enrichment of IL-2–STAT5 signaling in influenza-infected CD4^+^ T cells from *Prdm1*-CKO mice compared to WT mice (Fig. 3, C and D), suggesting that BLIMP1 repressed IL-2 signaling in influenza-infected CD4^+^ T cells. The genes associated with IL-2–STAT5 signaling exhibited greater expression levels in CD4^+^ T cells from *Prdm1*-CKO mice compared to those from WT mice (Fig. 3E).

As noted above, the CRISPR knockout screening was performed in an ATL-derived cell line, ED40515(+) cells. Because ATL cells typically have a T_reg_-like suppressor phenotype (*45*, *46*) and since the absence of BLIMP1 resulted in enhanced IL-2 signaling in cT_reg_ cells during influenza infection, we next investigated the role of BLIMP1 in IL-2 signaling in ex vivo–isolated T_reg_ cells. Splenic T_reg_ cells from WT (Foxp3^YFP-cre^) and *Prdm1*-CKO (*Prdm1*^fl/fl^Foxp3^YFP-cre^) mice were isolated, preactivated with anti-CD3 + anti-CD28, rested overnight, cultured with IL-2 for 24 hours, and then subjected to RNA-seq analysis (Fig. 3F). The GO analysis showed that the cytokine-mediated signaling pathway was differentially regulated in T_reg_ cells from *Prdm1*-CKO versus WT mice (Fig. 3G, red arrow). In the Hallmark pathway analysis, IL-2–STAT5 signaling was enriched with higher expression of genes associated with this pathway in T_reg_ cells from *Prdm1*-CKO mice (Fig. 3H, red arrow, and Fig. 3I). Thus, BLIMP1 limits IL-2–STAT5 signaling in ex vivo–isolated splenic T_reg_ cells as well as in influenza-infected mouse CD4^+^ T cells.

### Diminished BLIMP1 augments IL-2 signaling but attenuates T_reg_ suppressive activity

**Fig. 4.**
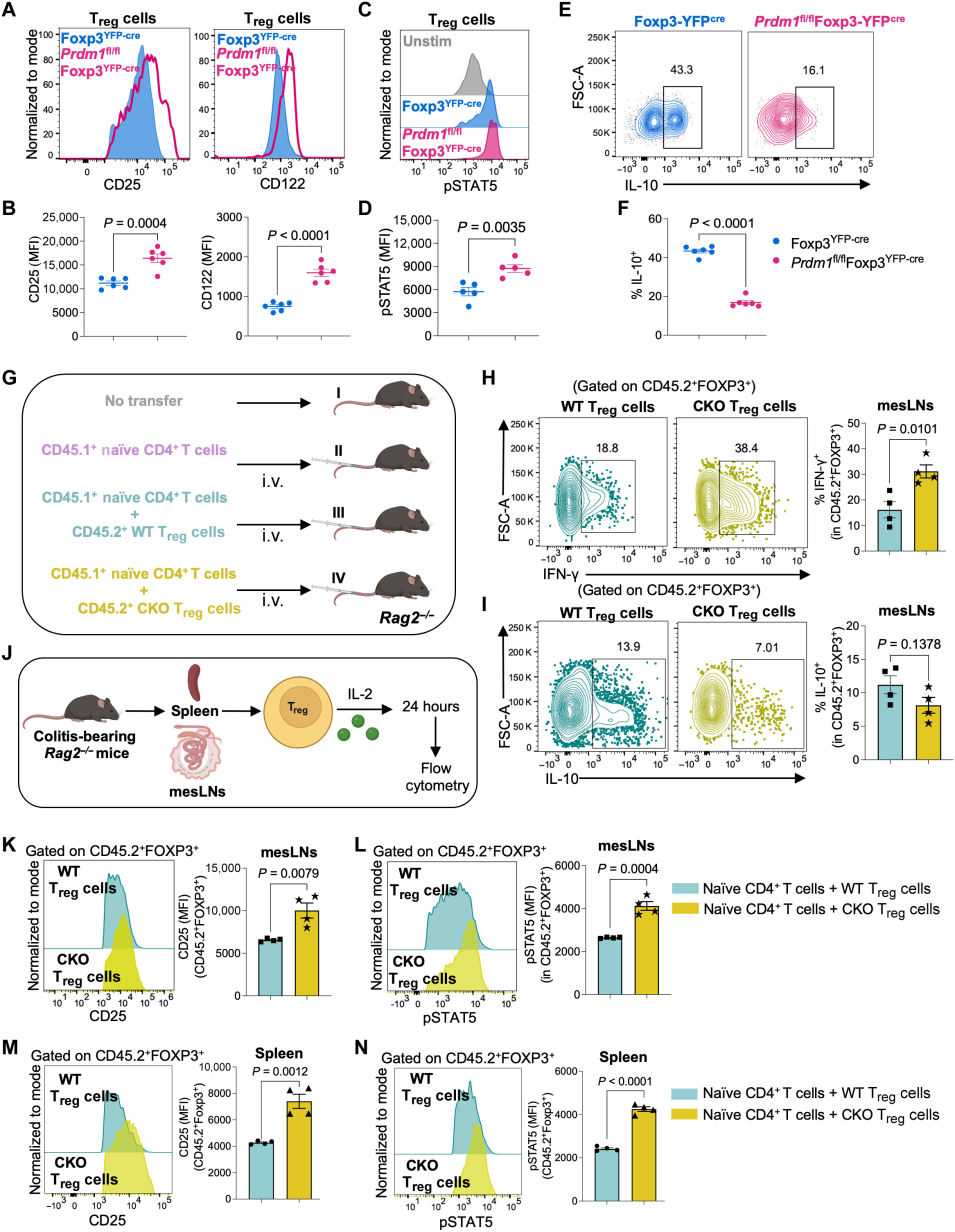
Absence of BLIMP1 augments IL-2 signaling and attenuates T_reg_ suppressive activity. (**A** to **F**) T_reg_ cells were purified from the spleens of Foxp3^YFP-cre^ (WT) and *Prdm1*^fl/fl^Foxp3^YFP-cre^ (*Prdm1*-CKO) mice. Cells were TCR-stimulated in the presence of IL-2 (500 IU/ml) for 72 hours at 37°C, harvested, and stained for flow cytometry analysis. Live cells were gated as CD4^+^CD25^+^FOXP3^+^ T_reg_ cells. Histograms and statistical analysis of protein expression for (A and B) CD25 and CD122, [(C) and (D)] pSTAT5, and [(E) and (F)] IL-10 by flow cytometry. Data are representative of means ± SEM from three independent experiments (*n* = 5 to 6 individual mice per group). A two-tailed unpaired Student’s *t* test was used for statistical analysis. (**G** to **N**) Adoptive T_reg_ transfer from Foxp3^YFP-cre^ (WT) or *Prdm1*^fl/fl^Foxp3^YFP-cre^ (*Prdm1*-CKO) mice to *Rag2^−/−^* mice with T cell transfer induced colitis. (G) Schematic representation for group I, *Rag2^−/−^* mice with no T cell transfer; group II, *Rag2^−/−^* mice with intravenous (i.v.) transfer of CD45.1^+^ naïve CD4^+^ T cells; group III, *Rag2^−/−^* mice with intravenous transfer of CD45.1^+^ naïve CD4^+^ T cells and CD45.2^+^ WT T_reg_ cells from WT mice; and group IV, *Rag2^−/−^* mice with intravenous transfer of CD45.1^+^ naïve CD4^+^ T cells and CD45.2^+^ CKO T_reg_ cells from *Prdm1*-CKO mice. Created in BioRender. S. Roy (2025) https://BioRender.com/debaxae. [(H) and (I)] Flow cytometry analysis for (H) IFN-γ and (I) IL-10 production by CD45.2^+^FOXP3^+^ T_reg_ cells in the mesLNs. (J) Schematic representation for ex vivo IL-2 stimulation of T_reg_ cells purified from spleen and mesLNs of *Rag2^−/−^* mice after colitis induction. Created in BioRender. S. Roy (2025) https://BioRender.com/debaxae. [(K) and (L)] Flow cytometry analysis of protein expression for (K) CD25 and (L) pSTAT5 in CD45.2^+^FOXP3^+^ T_reg_ cells from the mesLNs. [(M) and (N)] Flow cytometry analysis of protein expression for (M) CD25 and (N) pSTAT5 in CD45.2^+^FOXP3^+^ T_reg_ cells from the spleen. Two-tailed unpaired Student’s *t* test was used for statistical analysis. Data are representative of mean ± SEM from two independent experiments (*n* = 4 independent mice per group).

We further assessed the effects of BLIMP1 on IL-2 signaling in ex vivo–isolated FOXP3-YFP^+^ T_reg_ cells at the protein level (gating strategy shown in fig. S4A) and found that *Prdm1-*deficient T_reg_ cells had increased CD25 and CD122 expression (Fig. 4, A and B). Consistent with this, pSTAT5 was elevated in *Prdm1-*deficient T_reg_ cells (Fig 4, C and D); however, there were no significant changes in the levels of pAKT and pERK (fig. S4, B and C) in keeping with the observation that T_reg_ cells have defective PI3K/AKT signaling, which may explain their hypoproliferative response to IL-2 (*41*). We also found that IL-10 production was lower (Fig. 4, E and F), while FOXP3 and HELIOS expression were higher in *Prdm1-*deficient T_reg_ cells (fig. S4, D and E).

To complement these data, we assessed the in vivo functional relevance of *Prdm1* deletion in T_reg_ cells using a T cell–adoptive transfer colitis mouse model. Colitis was induced in *Rag2^−/−^* mice by intravenously injecting CD45.1^+^ naïve CD4^+^ T cells with or without cotransferring CD45.2^+^ T_reg_ cells from WT or *Prdm1*-CKO mice (Fig. 4G). There was a gradual decline in the body weight and shortening of the colon length in the mice that received the *Prdm1-*KO T_reg_ cells (group IV), similar to the colitis induced in mice that did not receive T_reg_ cells (group II); in contrast, the mice that received WT T_reg_ cells (group III) progressively gained weight (fig. S5, A and B). The colon histology images and scores indicated severe colitis in the mice receiving *Prdm1-*KO T_reg_ cells (group IV), while transfer of WT T_reg_ cells (group III) suppressed the development of colitis (fig. S5C). Mesenteric lymph nodes (mesLNs) and spleen were analyzed by flow cytometry for CD45.1^+^Tbet^+^ and CD45.2^+^FOXP3^+^ populations (fig. S5D). Colitis was not suppressed in the mice receiving *Prdm1-*KO T_reg_ cells, with greater accumulation of CD45.1^+^Tbet^+^IFN-γ^+^ T cells in the mesLNs and spleen than in mice that received WT T_reg_ cells (fig. S5, E to H). The increased intestinal inflammation in the mice receiving *Prdm1-*KO T_reg_ cells was associated with higher levels of IFN-γ and lower levels of IL-10 production in the CD45.2^+^FOXP3^+^ T_reg_ cells in the mesLNs (Fig. 4, H and I) and spleen (fig. S5, I and J). Furthermore, ex vivo stimulation with IL-2 induced higher expression of CD25 and pSTAT5 in CD45.2^+^FOXP3^+^ T_reg_ cells from the mesLNs (Fig. 4, K and L) and spleen (Fig. 4, M and N) from mice that received adoptively transferred *Prdm1-*CKO T_reg_ cells as compared to WT T_reg_ cells. These data indicate that in the absence of BLIMP1, T_reg_ cells lose their ability to suppress inflammatory responses in the intestine, which could be attributed to exacerbated IL-2 signaling and reduced IL-10 production, accompanied by heightened T helper 1 (T_H_1) effector responses with increased IFN-γ production.

### BLIMP1 restrains IL-2 signaling in human CD4^+^ T cells and T_reg_ cells

**Fig. 5.**
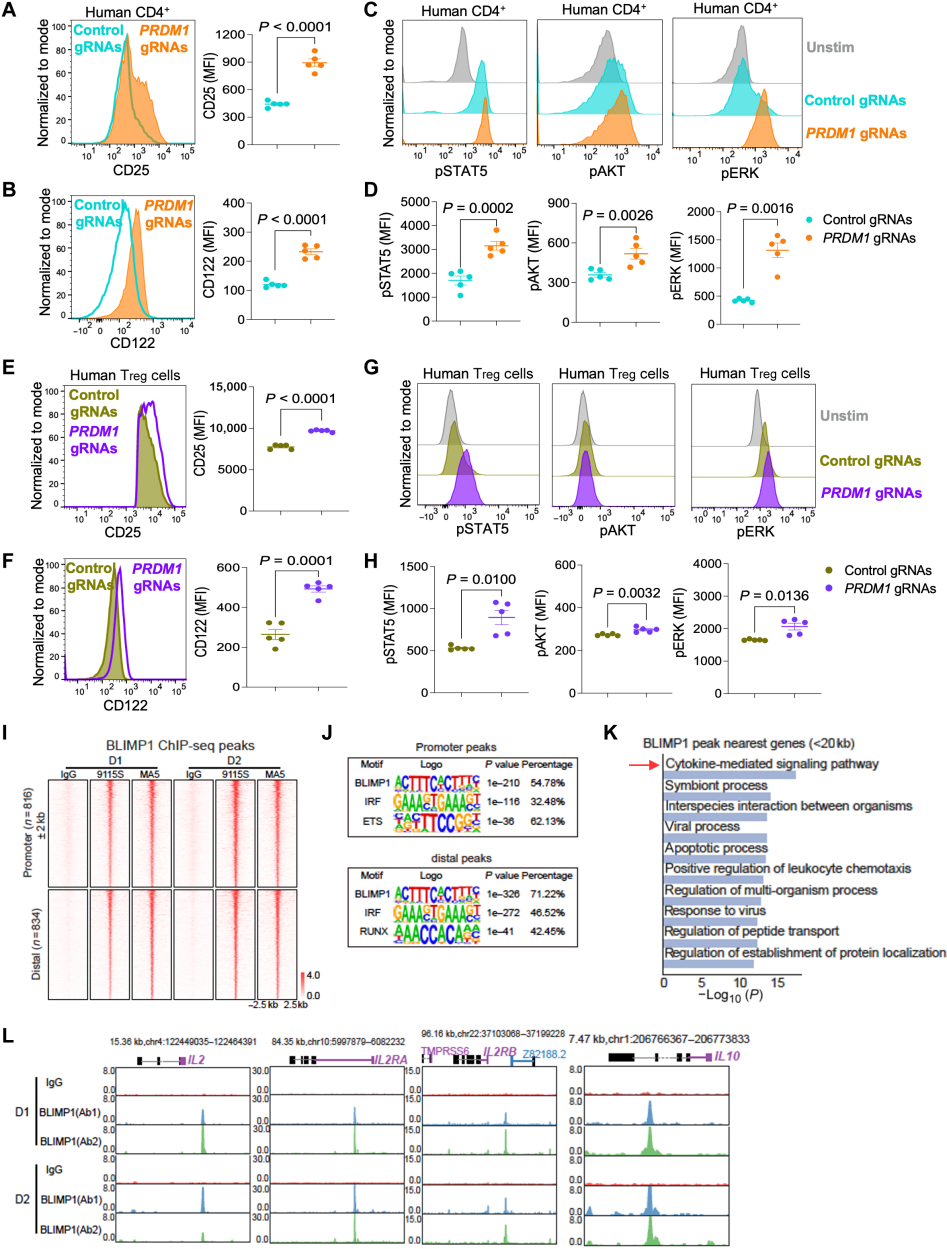
BLIMP1 restrains IL-2 signaling in human CD4^+^ T cells and natural T_reg_ cells. (**A** to **D**) Preactivated human CD4^+^ T cells were electroporated with control gRNAs or *PRDM1* gRNAs annealed with Cas9 as RNP complex, cultured with IL-2 (200 IU/ml) for 72 hours, and stained for flow cytometry. Histograms with statistical representation showing (A) CD25 and (B) CD122 protein levels by flow cytometry. (C) Histograms representing pSTAT5, pAKT, and pERK, staining by flow cytometry. (D) Statistical representation of flow cytometry analysis of pSTAT5, pAKT, and pERK. Data are representative of means ± SEM from three independent experiments (*n* = 5 individuals). Two-tailed paired Student’s *t* test was used for statistical analysis. (**E** to **H**) Preactivated human natural T_reg_ cells were electroporated with control gRNAs or *PRDM1* gRNAs annealed with Cas9 as RNP complex, cultured with IL-2 (500 IU/ml) for 72 hours, and stained for flow cytometry. Histograms with statistical representation showing (E) CD25 and (F) CD122 protein levels by flow cytometry. (G) Histograms representing pSTAT5, pAKT, and pERK staining by flow cytometry. (H) Statistical representation of flow cytometry analysis of pSTAT5, pAKT, and pERK. Data are representative of means ± SEM from three independent experiments (*n* = 5 individuals). Two-tailed paired Student’s *t* test was used for statistical analysis. (**I** to **L**) Natural T_reg_ cells from HDs (D1 and D2) were in vitro expanded in the presence of IL-2 followed by ChIP-seq analysis of BLIMP1 binding using antibodies BLIMP1 Ab1 (clone #9115S) and BLIMP1 Ab2 (clone #MA5-14879) as compared to the rabbit IgG control. (I) BLIMP1 ChIP-seq heatmaps (promoter versus distal regions). (J) Motif analysis for BLIMP1 peaks in the promoter and distal regions. (K) GO term enrichment for BLIMP1 peaks near TSS target genes. (L) Genome browser tracks showing BLIMP1-binding profiles at the indicated genes.

We next assessed whether the findings in mouse CD4^+^ T cells extended to human CD4^+^ T cells by using CRISPR-Cas9 gene editing to delete *PRDM1* in preactivated CD4^+^ T cells isolated from buffy coats of healthy donors (HDs). The gene editing efficiency in deleting the *PRDM1* gene was validated by performing a T7 endonuclease I assay in human CD4^+^ T cells (fig. S6A), and correspondingly, there was significantly reduced BLIMP1 protein in cells electroporated with *PRDM1*–guide RNAs (gRNAs) as compared to cells receiving control gRNAs (fig. S6B). CRISPR-Cas9–mediated deletion of *PRDM1* in human CD4^+^ T cells led to higher expression of CD25 and CD122 protein (Fig. 5, A and B) and a significant increase in pSTAT5, pAKT, and pERK (Fig. 5, C and D). As expected, there was also enhanced production of IL-2 in *PRDM1*-deleted human CD4^+^ T cells (fig. S6C).

We next examined the role of BLIMP1 in IL-2 signaling in natural T_reg_ cells isolated from buffy coats of healthy individuals by deleting *PRDM1* with CRISPR-Cas9. We again confirmed the knockout efficiency by the T7 endonuclease I assay in human T_reg_ cells (fig. S6D). BLIMP1 protein expression was markedly lower in the cells that received *PRDM1* gRNAs than in the cells that received control gRNAs (fig. S6E). We found higher levels of CD25 and CD122 protein expression after *PRDM1* deletion in human T_reg_ cells (Fig. 5, E and F). Consistent with the findings in mouse T_reg_ cells, *PRDM1* deletion in human T_reg_ cells also induced more pSTAT5 but only minimal changes in the expression of pAKT and pERK (Fig. 5, G and H). The protein expression levels of FOXP3 (fig. S6F) and HELIOS (fig. S6G) were elevated, while IL-10 production was diminished (fig. S6H) after *PRDM1* deletion in human T_reg_ cells. These results in the human CD4^+^ T cells and T_reg_ cells recapitulated those in the mouse, establishing that BLIMP1 restrains IL-2 signaling in CD4^+^ T cells, particularly T_reg_ cells, in both mice and humans.

To gain mechanistic insights into the regulation of IL-2 signaling by BLIMP1, we performed BLIMP1 chromatin immunoprecipitation sequencing (ChIP-seq) in human natural T_reg_ cells. Natural T_reg_ cells were isolated from healthy human donors, expanded in vitro in the presence of IL-2 and subjected to ChIP-seq analysis. BLIMP1 binding using two antibodies: BLIMP1 Ab1 (clone #9115S) and BLIMP1 Ab2 (clone #MA5-14879) was assessed as compared to rabbit anti–immunoglobulin G (IgG) control. The identification of BLIMP1 peaks revealed notably enriched genomic loci, visualized as intensity signals on the heatmaps. Approximately 50% of the BLIMP1 ChIP-seq peaks were localized within 2 kb of 5′ of the transcription start site (TSS) and thus were defined as binding in the promoter region, while the peaks more than 2 kb from the TSS were defined as distal regions (Fig. 5I). We performed de novo motif analysis to identify and explain the differential BLIMP1 binding and chromatin accessibility at the ChIP-seq peaks. The analysis revealed similar and highly enriched BLIMP1-binding motifs in both the promoter and distal regions; interferon regulatory factor (IRF) and E26 transformation-specific (ETS) were the second and third most significant motifs in the promoter region, and IRF and Runt-related transcription factor (RUNX) motifs were the second and third most significant in the distal regions (Fig. 5J). Overall, there were 1041 genes with BLIMP1 motifs, 526 with IRF motifs, 860 with ETS motifs, and 378 with RUNX motifs (table S1). BLIMP1 and IRF motifs were found for *IL2*, *IL2RA*, and *IL10*; ETS motifs for *IL2RA* and *IL10*; and RUNX motifs for *IL2RA*, indicating that more than one motif was identified for some key genes and suggesting that there may be cooperative effects among IRF, ETS, and RUNX family proteins with BLIMP1, an area for future investigation. GO analysis of BLIMP1 binding peaks proximal to TSS of target genes revealed enrichment of the cytokine-mediated signaling pathway, evidenced by the most significant *P* value (Fig. 5K, red arrow). BLIMP1 binds near the promoter regions of *IL2* and *IL2RB* but in the enhancer regions of *IL2RA* and *IL10* (Fig. 5L). Since BLIMP1 negatively regulates expression of IL-2, IL-2Rα, and IL-2Rβ, these findings are consistent with direct negative regulation of *IL2*, *IL2RA*, and *IL2RB* by BLIMP1. The binding to the *IL10* gene is interesting given that BLIMP1 is a positive regulator of IL-10 expression. Together, the ChIP-seq analysis and our functional data suggest that BLIMP1 directly binds and regulates the key genetic elements involved in IL-2 signaling in human T_reg_ cells.

### CD4^+^ T cells from patients with ATL have amplified IL-2 signaling

**Fig. 6.**
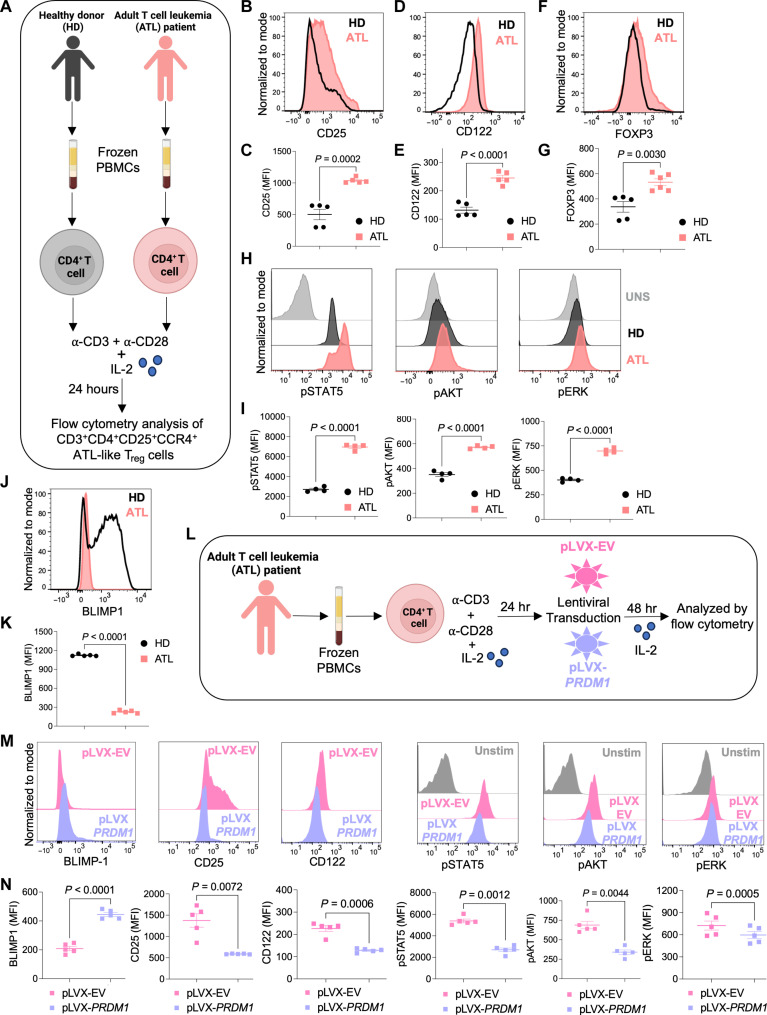
Patients with acute ATL showed augmented IL-2 signaling as compared to HDs. Purified CD4^+^ T cells from frozen PBMCs of HDs and patients with acute ATL were ex vivo stimulated with anti-CD3 and anti-CD28 and IL-2 for 24 hours at 37°C and stained for flow cytometry. Cells were gated as CD3^+^ CD4^+^ CD25^+^ CCR4^+^ T cells (ATL-like T_reg_ cells). (**A**) Schematic representation of the experiment (**B** to **K**). Created in BioRender. S. Roy (2025) https://BioRender.com/ae8e1h0. Representative histograms with cumulative statistics for flow cytometry analysis of [(B) and (C)] CD25 and [(D) and (E)] CD122 expression. [(F) and (G)] Representative histograms and cumulative statistics for FOXP3 expression by flow cytometry. [(H) and (I)] Flow cytometric analysis of pSTAT5, pAKT, and pERK showing representative histograms and cumulative statistics. [(J) and (K)] Representative histograms and statistics for BLIMP1 expression by flow cytometry. Data are representative of means ± SEM from three independent experiments (*n* = 4 to 5 individuals). Two-tailed unpaired Student’s *t* test was used for statistical analysis. (**L**) Schematic representation of *PRDM1* overexpression in ATL cells. Created in BioRender. S. Roy (2025) https://BioRender.com/ae8e1h0. (**M**) Histograms showing protein expression for BLIMP1, CD25, CD122, pSTAT5, pAKT, and pERK in ATL cells transduced with pLVX-EV (empty vector control) or pLVX-*PRDM1* (vector expressing *PRDM1*). (**N**) Statistical analysis showing mean fluorescent intensity (MFI) for BLIMP1, CD25, CD122, pSTAT5, pAKT, and pERK, protein levels in ATL cells transduced with pLVX-EV (empty vector control) or pLVX-*PRDM1* (vector expressing *PRDM1*). Data are representative of means ± SEM from two independent experiments (*n* = 5 individuals). Two-tailed paired Student’s *t* test was used for statistical analysis.

The results in mouse cells, including influenza infection and T cell–induced colitis models, as well as in primary human cells, indicate that BLIMP1 negatively regulates IL-2 signaling that helps to modulate the immune response. Since the CRISPR knockout screening was performed in an IL-2–dependent ATL cell line, we next analyzed primary cells from patients with acute ATL. Purified CD4^+^ T cells from frozen peripheral blood mononuclear cells (PBMCs) of HDs and patients with acute ATL were stimulated with anti-CD3 + anti-CD28 and IL-2 for 24 hours at 37°C (Fig. 6A). Cells were gated as CD3^+^CD4^+^ CD25^+^CCR4^+ “^ATL-like T_reg_” cells [based on the phenotype of ATL cells with fluorescence minus one, (FMO) controls] and analyzed by flow cytometry (fig. S7, A to D). As expected, the frequency of these cells were much lower in HDs, while most ATL cells had this phenotype (fig. S7D). T_reg_-like cells from patients with ATL showed higher expression of CD25 and CD122 as compared to HDs (Fig. 6, B to E). Consistent with ATL cells having T_reg_-like suppressor activity (*45*, *46*), there was higher FOXP3 expression in cells from patients with ATL than in HDs (Fig. 6, F and G). We also found more IL-10–producing T_reg_-like cells in patients with ATL in comparison to HDs, consistent with the suppressive properties of ATL cells (fig. S7E). Cells from patients with ATL showed augmented IL-2 signaling as manifested by enhanced phosphorylation of STAT5, AKT, and ERK (Fig. 6, H and I). Notably, BLIMP1 protein expression was lower in cells ex vivo treated with IL-2 from patients with ATL as compared to HDs either after 24 hours (Fig. 6, J and K) or 48 or 72 hours (fig. S7, F and G), inversely correlating BLIMP1 expression with activated IL-2 signaling in cells from patients with acute ATL. We also investigated other potential regulators of IL-2 signaling such as Tbet, PTEN, and THEMIS (*3*, *41*, *47*). We observed a modest increase in expression of PTEN but decreased expression of Tbet and THEMIS in ATL cells as compared to HDs (fig. S7, H and I), suggesting their possible role in ATL; however, the impact of BLIMP1 was clearly more evident.

Because the above data indicate that the activated IL-2 signaling in T cells from patients with acute ATL was associated with lower BLIMP1 protein levels, we further investigated a mechanistic role for BLIMP1 by transducing either control or *PRDM1-*expressing lentiviruses in ATL cells and examining the effect of IL-2 (Fig. 6L). Increasing BLIMP1 expression in ATL cells resulted in significantly lower CD25 and CD122 protein levels and decreased phosphorylation of STAT5, AKT, and ERK (Fig. 6, M and N). These results suggest that BLIMP1 expression reciprocally regulates the activation of IL-2 signaling in patients with acute ATL.

### *PRDM1* expression inversely correlates with IL-2 signaling in patients with ATL

**Fig. 7.**
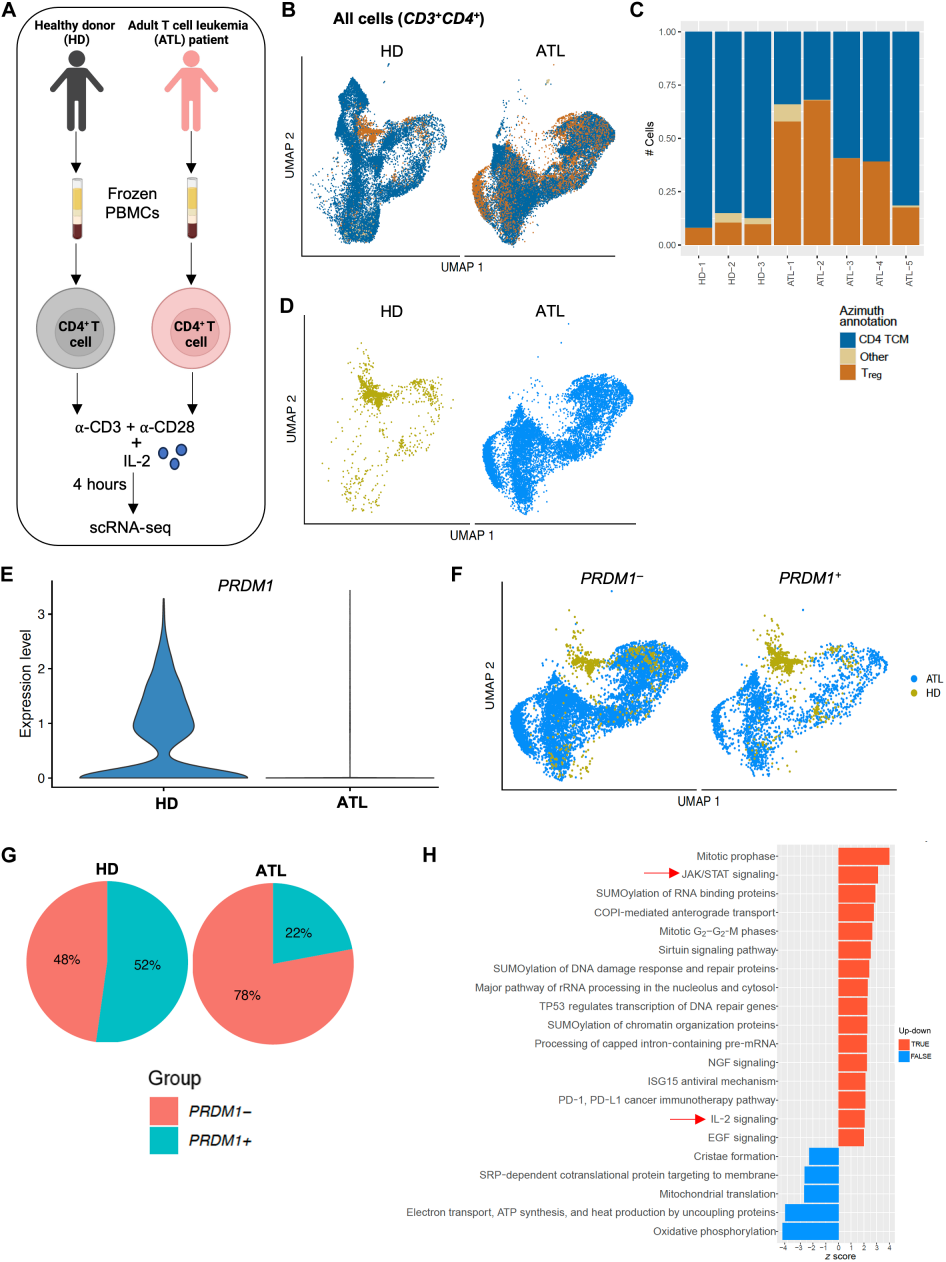
*PRDM1* expression inversely correlates with activated IL-2 signaling in patients with acute ATL. Purified CD4^+^ T cells from HDs (*n* = 3) and patients with acute ATL (*n* = 5) were stimulated with anti-CD3 and anti-CD28 in presence of IL-2 for 4 hours at 37°C. The cells were harvested and subjected to scRNA-seq. (**A**) Schematic representation of the experimental setup for (**B** to **H**). Created in BioRender. S. Roy (2025) https://BioRender.com/9zdi679. (B) Uniform Manifold Approximation and Projection (UMAP) plot showing all cells split by HD cells (left) and ATL cells (right). (C) Bar plot showing proportion of the cells in each cluster annotated by Azimuth for each HD and ATL sample. (D) UMAP plot showing distribution of T_reg_ cells split by HD cells (left) and ATL cells (right). (E) Violin plot showing *PRDM1* expression in T_reg_ cells in HDs and patients with ATL. (F) UMAP plot showing distribution of *PRDM1^−^* versus *PRDM1^+^* T_reg_ populations in HDs and patients with ATL. (G) Pie chart showing percentage of cells in HDs and patients with ATL that express any amount of *PRDM1*. (H) Bar plot showing top pathways (by *z* score) from Qiagen IPA using differentially expressed genes derived from *PRDM1^−^* versus *PRDM1^+^* populations within T_reg_ cells cluster in patients with ATL.

We next performed single-cell RNA-seq (scRNA-Seq) analyses in CD4^+^ T cells from HDs and patients with acute ATL that were stimulated with anti-CD3 and anti-CD28 in the presence of IL-2 for 4 hours at 37°C (Fig. 7A). An integrated scRNA-seq dataset from three HD samples and five ATL samples was annotated with Azimuth. The CD4^+^ T cells were annotated as “CD4 TCM” or central memory T cells (*n* = 26978), “T_reg_” cells (*n* = 11455), and “Other” T cells (*n* = 404) (which was a mix of cells annotated as CD4^+^ proliferating, naïve, and effector memory cells) (Fig. 7B) (see table S2 for the markers used by Azimuth to define the populations). All five ATL samples showed a higher proportion of T_reg_ cells compared to the three HD samples (Fig. 7C). An integrated dataset for T_reg_ cells from the five ATL and three HD samples showed higher abundance of T_reg_ cells in ATL as compared to HD (Fig. 7D). Unsupervised clustering revealed seven different subpopulations (clusters 0 to 6) of T_reg_ cells (fig. S7J). The T_reg_ population in ATL samples was more diverse compared to HD samples (fig. S7J). More than 70% of T_reg_ cells in HD samples were clustered together in cluster 4, which was nearly absent in ATL. This suggested the presence of one major T_reg_ subpopulation in HD samples, whereas ATL samples had multiple T_reg_ subpopulations (fig. S7, J and K). The seven subclusters of T_reg_ cells in HD and ATL showed differential gene expression profile with top 32 genes average expression being represented (fig. S7L). We next evaluated *PRDM1* expression in T_reg_ cells and found higher *PRDM1* expression in HD as compared to ATL (Fig. 7E). The integrated dataset from HD and ATL showed a higher proportion of *PRDM1^−^* versus *PRDM1^+^* subpopulations in T_reg_ cells in ATL but almost equal proportions of *PRDM1^−^* and *PRDM1^+^* subpopulations in T_reg_ cells in HD (Fig. 7F). Approximately 78% of T_reg_ cells in ATL were *PRDM1^−^*, whereas the *PRDM1^+^* and *PRDM1^−^* subpopulations were almost equivalent in HD (Fig. 7G). Thus, most T_reg_-like cells in ATL lack *PRDM1* expression. Using the differentially expressed genes (DEGs) as input (see table S3), the Qiagen Ingenuity Pathway Analysis (IPA) in *PRDM1^−^* versus *PRDM1^+^* populations within T_reg_ cells revealed up-regulation of pathways including JAK-STAT signaling and IL-2 signaling pathways in the *PRDM1^−^* subpopulation of cells from patients with ATL (Fig. 7H; see table S4 for the genes used by the Qiagen IPA to identify the pathways). This suggests that diminished *PRDM1* expression potentially contributes to the constitutively activated JAK-STAT signaling and IL-2 signaling that are often found in ATL cells (*40*).

## DISCUSSION

The immunomodulatory effects of IL-2 on both T_EFF_ cells and T_reg_ cells make it a promising target for immunotherapy (*48*). IL-2 has been exploited to selectively expand T_reg_ cells in autoimmune diseases, while in cancer, expanding T_EFF_ cells is desirable (*49*–*54*). Considering the immunostimulatory and immunosuppressive consequences of IL-2 signaling in different diseases, a deep understanding of its regulation is important. We therefore investigated IL-2 signaling by performing a genome-wide CRISPR knockout screen using IL-2–dependent ED40515(+) cells derived from patients with HTLV-I–associated ATL.

The screen showed a dropout of sgRNAs for the genes *IL2RA*, *IL2RB*, *JAK3*, and *STAT5B*, which are required for IL-2 signaling, validating the screen. In contrast, all four sgRNAs for both *PRDM1* and *PTEN* were significantly enriched. PTEN is a known inhibitor of IL-2R signaling in T_reg_ cells where it inhibits PI3K-AKT signaling and IL-2–mediated cell proliferation, while the JAK-STAT5 signaling remains intact attributing to the hypoproliferative phenotype of T_reg_ cells in response to IL-2 (*41*, *42*). BLIMP1 (encoded by *PRDM1* in human and *Prdm1* in mouse) is a transcription factor that inhibits IL-2 production by binding directly to the *Il2* promoter in T cells repressing the cell proliferation (*24*, *35*). In turn, IL-2 induces BLIMP1 expression, establishing a negative feedback loop limiting its own production (*24*, *35*). It is known that IL-2 primarily acts on the responding cells in a paracrine fashion by inducing phosphorylation of STAT5 (*55*, *56*). Our CRISPR knockout screen implicated BLIMP1 as a negative regulator of IL-2–mediated proliferation. Here, we demonstrated that the overexpression of *Prdm1* in mouse CD4^+^ T cells inhibited IL-2 signaling, whereas deletion of *Prdm1* in mouse CD4^+^ T cells resulted in enhanced IL-2 signaling, as evidenced by increased protein expression of CD25 and CD122 as well as increased phosphorylation of STAT5, AKT, and ERK. These findings are also consistent with higher expression of *Il2ra* and *Il2rb* mRNA expression in LCMV-infected CD8^+^ T cells from *Prdm1*-knockout mice (*57*), although IL-2 signaling was not examined in that study. Previously, BLIMP1-deficient CD8^+^ T cells were shown to have an impaired effector response to influenza virus (*43*). IL-2 signaling has been shown to be highest at the peak of influenza infection in various CD4^+^ T cell populations, such as T_FH_, T_FR_, T_EFF_, and T_reg_ cells (*44*). Here, we demonstrate that BLIMP1 deficiency results in augmented IL-2 signaling in T_FH_, T_FR_, T_EFF_, and cT_reg_ cells after infection with influenza virus, with the most robust effect in the T_reg_ cells. Viral antigen has been shown to induce the T_reg_ cells in influenza virus–infected mice (*58*); in mice with BLIMP1-deficient T cells, following influenza infection, there was increased frequency of T_reg_ cells and T_EFF,_ with decreased IL-10 but enhanced IFN-γ production and increased IL-2 signaling in medLNs and lungs. This creates a dysregulated microenvironment that is ineffective in controlling the inflammatory responses in the medLNs and lungs of influenza virus–infected mice that succumb to the deleterious effects of augmented IL-2 signaling as well as the abrogated IL-10 production. This is consistent with a known positive regulation of IL-10 gene expression by BLIMP1 (*59*), in addition to negative effects of BLIMP1 on IL-2 production and IL-2 signaling as we show in this study. Thus, BLIMP1 is critical in modulating effector and regulatory functions of T_EFF_ and T_reg_ cells during influenza virus infection.

Selectively knocking out *Prdm1* in Foxp3-expressing T_reg_ cells resulted in higher IL-2 signaling, with increased expression of T_reg_ transcription factors FOXP3 and HELIOS but reduced IL-10 production. Moreover, adoptive transfer of *Prdm1*-deficient T_reg_ cells into *Rag2^−/−^* mice failed to suppress the T cell–induced colitis, likely due to their defective IL-10 production (*60*–*63*). In the absence of BLIMP1, transferred CD45.2^+^FOXP3^+^ T_reg_ cells acquired an effector phenotype, producing more IFN-γ but less IL-10, which attenuates their ability to suppress the colonic intestinal inflammation. It is known that increased IFN-γ production after adoptive transfer of naïve CD4^+^ T cells into *Rag2*^−/−^ mice contributes to the development of colitis (*64*, *65*). In addition, a prior study showed that Tbet, which induces IFN-γ from T_reg_ cells, contributes to colitis (*66*). IL-2 can induce Tbet and IL-12Rβ2 and promote T_H_1 differentiation with enhanced IFN-γ production (*67*), indicating a mechanism for how increased IL-2 signaling may enhance IFN-γ production in T_reg_ cells and limit their suppressive function, observations that support the idea that IFN-γ derived from BLIMP1-deficient T_reg_ cells may contribute to inflammation in colitis and possibly in other autoimmune conditions. Furthermore, the enhanced IL-2 signaling in the transferred *Prdm1*-knockout T_reg_ cells likely contributed to the T cell–induced colitis by expanding the number or function of these poorly suppressive “effector” T_reg_ cells. Although the contribution of IL-2 signaling in colitis is unclear, the increased IL-2 signaling due to diminished BLIMP1 potentially provides an additional explanation for the aggravated inflammation during colitis in addition to the effects of lower levels of IL-10 and CTLA-4 (*60*–*63*). Consistent with this idea, a recent study reported that the duplication of *IL2RA* locus results in excessive IL-2 signaling and that this predisposed to colitis in patients with inflammatory bowel disease (*68*). Other studies have shown that overexpression of CD25 occurs in various autoimmune diseases and that blocking it can improve inflammation and clinical outcome (*69*, *70*). Thus, optimal BLIMP1 expression is vital for maintaining balanced IL-2 signaling and intestinal homeostasis.

In addition to augmented IL-2 signaling after *Prdm1* deletion in the mouse, *PRDM1* deletion also resulted in increased IL-2 signaling in human CD4^+^ T cells and natural T_reg_ cells. ChIP-seq showed binding of BLIMP1 to key genes associated with IL-2 signaling in human T_reg_ cells. Since IL-2 activates JAK1 and JAK3 (*71*), JAK inhibitors including ruxolitinib and tofacitinib has been used for the treatment of immunosuppressive diseases (*72*). The combination of ruxolitinib and navitoclax (an inhibitor of Bcl2/Bcl-xL) showed promising antitumor efficacy and prolonged survival in a mouse model of ATL (*73*). Although combination chemotherapy and immunotherapy has been used in the treatment of ATL, long-term success has been very limited, indicating a need for new therapeutic strategies. Acute ATL is the most common and aggressive form of ATL and is characterized by clonal expansion of HTLV-1–infected cells. Here, we demonstrated that patients with acute ATL have enhanced IL-2 signaling with reciprocally lower BLIMP1 expression and that overexpression of BLIMP1 could repress IL-2 signaling. scRNA-seq analysis established that *PRDM1* expression inversely correlates with the activation of JAK-STAT5 and IL-2 signaling in ATL-like T_reg_ cells from patients with acute ATL. Consequently, diminished induction of BLIMP1 correlates with augmented JAK-STAT and IL-2 signaling in ATL. In summary, we propose here that BLIMP1 serves as a pivotal regulatory node in the IL-2 signaling pathway, and thus modulating the activity of BLIMP1 may allow better control of IL-2–mediated diseases by calibrating immune responses and maintaining immune homeostasis.

## METHODS

### Healthy human donors and patients

Buffy coats from healthy human volunteers were obtained from National Institutes of Health (NIH) Blood Bank, Bethesda, USA. Because Buffy coats are produced as a by-product of a volunteer whole-blood donation for transfusion and are thus already in existence (preexisting material), would otherwise be discarded if not distributed for research use, and are irreversibly anonymized before distribution, they meet the FDA 45 CFR46 2018 Revisions of the Common Rule and are exempt from the requirement for Institutional Review Board (IRB) review and informed consent. Frozen PBMCs from blood samples of patients with acute ATL were obtained from patients enrolled under a clinical trial approved by the NCI IRB, protocol number 97-C-0143. The original samples were collected under an approved protocol of the NCI. All cells provided to us were deidentified and were not considered human subjects research and did not require IRB approval. PBMCs from HDs were isolated and stored in liquid nitrogen to be used as controls. The information on the samples from patients with ATL is listed in table S5.

### Mice

C57BL/6 (B6) mice were obtained from the Charles River Laboratory (strain #027) and B6-CD45.1 mice (B6.SJL-*Ptprc^a^Pepc^b^*/BoyJ) were obtained from the Jackson laboratory (strain #002014). B6-*Rag2^−/−^* (C57BL/6-*Rag2^−/−^*) mice (*74*) were from Taconic. *Prdm1*^fl/fl^ mice (*75*, *76*) (the Jackson Laboratory strain #008100) were crossed with CD4^cre^ mice (the Jackson Laboratory, strain #022071) and Foxp3^YFP-cre^ mice (the Jackson Laboratory, strain #016959) to generate *Prdm1*^fl/fl^CD4^cre^ and *Prdm1*^fl/fl^Foxp3^YFP-cre^ mice, respectively. All mice were housed in pathogen-free BSL2 AALAC-accredited facilities at the NIH, Bethesda, USA. All mice used for experiments were 8 to 12 weeks old and both age and sex matched. All animal experiments were performed using protocols approved by the National Heart, Lung and Blood Institute (NHLBI) Animal Care and Use Committee and followed NIH guidelines for use of animals in intramural research.

### Mouse flow cytometry antibodies

The following antibodies were used to perform flow cytometry on mouse cells (all antibodies are from BioLegend unless indicated otherwise): CD4 (clone RM4-5), CD25 (clone PC-61), CD122 (clone TM-β1), CD19 (clone 6D5), CD45RB (clone C363-16A), CD45 (clone 30-F11), CD45.1 (clone A20), CD45.2 (clone 104), PD-1 (clone 29F.1A12), CXCR5 (clone L138D7), Bcl-6 (clone 7D1), Tbet (clone 4B10), BLIMP1 (clone 5E7), FOXP3 (clone MF-14), HELIOS (clone 22F6), IL-2 (clone JES6-SH4), IL-10 (clone JESS-16E3), IFN-γ (clone XMG1.2), live/dead near infrared (IR) (L10119, Thermo Fisher Scientific), pSTAT5 (clone 47/Stat5(pY694), BD Biosciences), pAKT (clone M89-61, BD Biosciences), and pERK (clone 6B8B69). The influenza PR8-specific NP311-325 tetramer conjugated to BV421 was provided by the NIH tetramer core facility.

### Human flow cytometry antibodies

The following antibodies were used to perform flow cytometry on human cells (all antibodies are from BioLegend unless indicated otherwise): anti-CD3 (clone OKT3), CD4 (clone OKT4), CD25 (clone BC96), CCR4 (clone L291H4), CD122 (clone TU27), live/dead near IR (L10119 Thermo Fisher Scientific), BLIMP1 (clone 6D3), FOXP3 (clone 150D), HELIOS (clone 22F6), IL-2 (clone MQ1-17H12), IL-10 (clone JES-9D7), Tbet (clone 4B10), THEMIS (clone REA463), PTEN (clone A2B1), pSTAT5 [clone 47/Stat5(pY694), BD Biosciences], pAKT (clone M89-61, BD Biosciences), and pERK (clone 6B8B69).

### Antibodies and reagents

The list of antibodies and reagents used in this study is provided in table S6.

### CRISPR-Cas9 knockout screening in ED40515(+) cell line

ED40515(+) cell line with stable Cas9 expression were generated as described (*77*). For sgRNA library construction, the human CRISPR pooled genome-wide Brunello sgRNA library (*78*) (Addgene, #73178) was cloned in pLKO-based sgRNA vector (Addgene, #52628) and transformed in *Stbl4* bacteria (Invitrogen). Lentiviruses were produced in 293FT cells by transfecting the sgRNA plasmid library with packaging vectors pCMV-VSV-G (Addgene, #8454) and pCMV-dR8.2 dvpr (Addgene, #8455) in a 10:1:10 ratio using Lipofectamine 3000 reagents according to the manufacturer’s instructions. Viral supernatants were harvested and concentrated using a Lenti-X concentrator (CloneTech), and the viral titer was determined by Lenti-X GoStix (Takara). ED40515(+) cells were transduced with the concentrated lentiviral supernatants by centrifugation at 3500 rpm for 45 min at 30°C, and the transduced cells were selected using puromycin (2 μg/ml) after 48 hours. Cells were transduced with a multiplicity of infection of 0.3 to achieve 500× coverage of the library after 5 days of puromycin selection. Cells were then cultured for 21 days in the presence of IL-2 (200 IU/ml; #Ro 23-6019, Roche) and doxycycline (200 ng/ml; #D5207, Sigma-Aldrich) to induce Cas9 expression. DNA was isolated on day 21 followed by library amplification and sequencing on Illumina NextSeq500 platform as described (*77*, *79*). An average of 150× (126 to 199×) sequencing depth was achieved, and data analyzed as described previously (*77*). Sequences were aligned to the sgRNA library, allowing for 1–base pair (bp) mismatch using custom scripts and Bowtie 2 version 2.2.9 with the following parameters: -p 16 -f --local -k 10 --very-sensitive-local -L 9 -N1. Pairwise differential sgRNA comparison between day 0 and day 21 was performed using R package edgeR. Differentially expressed sgRNAs were identified and displayed with volcano plots to show the biological difference (log_2_FC), and statistical significance [−log_10_FDR (false discovery rate)]. Positive selection [log_2_FC(D21/D0) > 0] and negative selection [log2FC(D21/D0) < 0] indicated enriched to depleted sgRNAs of potential interest.

### Mouse CD4^+^ T cell isolation and culture

CD4^+^ T cells were isolated from mouse spleens using a negative selection kit (#19852, Stem Cell Technologies) according to the manufacturer’s protocol. Purified CD4^+^ T cells were activated with plate-bound anti-mouse CD3 (2 μg/ml, #BE0001-1; clone 145-2C11, BioXCell) and soluble anti-mouse CD28 (1 μg/ml, #BE0015-1; clone 37.51, BioXCell) and cultured in RPMI 1640 medium (#11875093, Gibco) supplemented with 10% fetal bovine serum (FBS) (#100-106, GeminiBio), 2 mM l-glutamine (#25030-149, Gibco), penicillin (50 U/ml) + streptomycin (50 μg/ml) (#15070-063, Gibco), and 50 μM 2-mercaptoethanol (#21985-023, Gibco) for 72 hours in the presence of recombinant human IL-2 (100 IU/ml; #Ro 23-6019, Roche) at 37°C after which the cells were harvested and assayed.

### Mouse CD4^+^Foxp3^+^ T_reg_ isolation and culture

CD4^+^ T cells were isolated from mouse spleen and lymph nodes by negative selection as described above. Foxp3^+^ T_reg_ cells were sorted from enriched CD4^+^ T cells suspension based on YFP expression using a FACSAria cell sorter (BD Biosciences). Sorted CD4^+^Foxp3^+^ T_reg_ cells were activated using plate-bound anti-CD3 (2 μg/ml) and soluble anti-CD28 (1 μg/ml) and cultured in complete RPMI 1640 medium with recombinant human IL-2 (500 IU/ml) for 72 hours at 37°C following which the cells were harvested and analyzed.

### Human CD4^+^ T cell isolation and culture

PBMCs were isolated from buffy coats using lymphocyte separation medium (#MT25072CI, Corning) based on density gradient centrifugation. CD4^+^ T cells were purified from PBMCs by negative selection following the kit’s protocol (#17952, Stem Cell Technologies). CD4^+^ T cells were then activated with plate-bound anti-human CD3 (2 μg/ml, #BE0001-2; clone OKT3, BioXCell) and soluble anti-human CD28 (1 μg/ml; #BE0248; clone 9.3, BioXCell) and cultured in RPMI 1640 medium with 10% FBS, 2 mM l-glutamine, and penicillin (50 U/ml) + streptomycin (50 μg/ml) in the presence of recombinant human IL-2 (100 IU/ml) for 72 hours at 37°C.

### Human CD4^+^CD25^+^CD127^low^ natural T_reg_ isolation and culture

PBMCs were isolated from buffy coats of healthy human donors as described above. CD4^+^CD25^+^CD127^low^ natural T_reg_ cells were purified by magnetic separation according to the manufacturer’s instructions (#18063, Stem Cell Technologies). Purified natural T_reg_ cells were activated using plate-bound anti-CD3 (2 μg/ml) and soluble anti-CD28 (1 μg/ml) and then cultured in complete TexMACS medium (#130-097-197, Miltenyi Biotec) supplemented with 5% human AB serum (#100-812-100, GeminiBio), 2 mM l-glutamine, and penicillin (50 U/ml) + streptomycin (50 μg/ml) for 7 days at 37°C in the presence of recombinant human IL-2 (500 IU/ml). The media were replenished every alternate day containing recombinant human IL-2 (500 IU/ml).

### Retroviral transduction

The cDNA was polymerase chain reaction (PCR) amplified for *Prdm1* gene from purified mouse CD8^+^ T cells using forward (ATCGAGATCTATGAGAGAGGCTTATCTC) and reverse (CTAGAGATCTTTAAGGATCCATCGGTT) primers, cloned into pRV-EV (*80*), using restriction enzyme BglII (#RO144S, NEB) and transfected with the pCL-Eco retrovirus packaging plasmid (#12371, Addgene) into 293T cells. Retroviral supernatants carrying the pRV-EV (empty vector) or pRV-*Prdm1* (vector-expressing *Prdm1*) plasmids were mixed with polybrene (8 μg/ml; #TR-1003, Sigma-Aldrich) and transduced into preactivated mouse CD4^+^ T cells by centrifugation at 3500 rpm for 45 min at 30°C. The retroviral supernatants were replaced with new medium containing human recombinant IL-2 (100 IU/ml), and the cells were cultured for 2 days at 37°C and harvested for further analysis.

### CRISPR-Cas9 deletion of *PRDM1*

Four *PRDM1*-gRNA target sequences [*PRDM1* complementary RNA 1 (crRNA1), crRNA2, crRNA3, and crRNA4] and four control gRNA sequences (control crRNA1, crRNA2, crRNA3, and crRNA4) were selected from the Brunello library that was used for the CRISPR-Cas9 knockout screening (table S7) and purchased from Integrated DNA Technologies (IDT). First, 80 μM crRNA (IDT) and 80 μM trans-activating CRISPR RNA (tracrRNA) (#10007810, IDT) were mixed in a 1:1 ratio and incubated for 30 min at 37°C to generate 160 μM crRNA-tracrRNA duplexes (gRNAs). Cas9 nuclease (40 μM, #1081059, IDT) was slowly added to the crRNA-tracrRNA duplexes (gRNAs) and incubated for 15 min at 37°C to generate ribonucleoprotein (RNP) complexes. For each reaction, 10 × 10^6^ stimulated human CD4^+^ T cells or T_reg_ cells were pelleted and resuspended in 20 μl of P3 primary cell nucleofection solution (#V4XP-3032, Lonza) and 2 μl of Cas9-RNP complexes and 0.75 μl of 100 μM electroporation enhancer (#1075916, IDT) were added, and the entire volume was transferred to a nucleocuvette strip (Lonza). The cells were electroporated using the EH-115 program on a Amaxa 4D-nucleofector (Lonza), 80 μl of prewarmed RPMI 1640 medium was added to each well after electroporation, and the cells were allowed to recover for 30 min at 37°C. The cells were then cultured in TexMACS medium supplemented with 5% human AB serum, 2 mM l-glutamine, and penicillin (50 U/ml) + streptomycin (50 μg/ml) for 3 to 4 days at 37°C in the presence of IL-2 (200 IU/ml) for human CD4^+^ T cells and IL-2 (500 IU/ml) for human T_reg_ cells. Gene editing efficiency was estimated by performing a T7 endonuclease I assay (#M0302S, New England Biolabs) per the manufacturer’s protocol using the primers listed in table S8.

### Intracellular staining and flow cytometry

The cells were resuspended in staining buffer [phosphate-buffered saline (PBS) with 2% FBS and 0.02% sodium azide] and stained for live/dead using LIVE/DEAD Fixable Dead Cell Stain Kit (#L10119, Invitrogen) and surface markers for 20 min at room temperature. Cells were fixed and permeabilized with Cytofix/Cytoperm (#554714, BD Biosciences) for intracellular cytokine staining and Foxp3/transcription factor staining buffers (#00-5523-00, eBioscience) for intracellular staining of transcription factors according to the instructions in the kit. Before staining, cells were stimulated with phorbol 12-myristate 13-acetate (50 ng/ml; #P1585, Sigma-Aldrich), ionomycin (1 μg/ml; #13909, Sigma-Aldrich), and 1× of protein transport inhibitor (#00-4980-03, eBioscience) for 5 hours at 37°C. For pSTAT5, pAKT, and pERK staining, the cells were rested overnight in complete medium at 37°C and then stained for live/dead and stimulated with recombinant human IL-2 for 10 min at 37°C. The cells were immediately fixed in 2.5% paraformaldehyde (#043368-9 M, Thermo Fisher Scientific), permeabilized in 100% cold methanol (#326950010, Thermo Fisher Scientific), and stained with the respective surface markers and phospho flow antibodies for 1 hour at room temperature. After staining, the cells were acquired using a BD LSR Fortessa X-20 Cell Analyzer flow cytometer (BD Biosciences) and data were analyzed with FlowJo software (TreeStar). All the antibodies were from BD Biosciences or BioLegend.

### PR8 influenza infection mouse model

One thousand viral focal units of PR8 influenza virus recombinant strain [A/Puerto Rico/8/1934 (H1N1)] (*81*, *82*) in 100 μl of PBS was administered intranasally into *Prdm1*^fl/fl^ (WT) and *Prdm1*^fl/fl^CD4^cre^ (CKO) mice after anesthesia with ketamine/xylazine. Infection was allowed to develop for 10 days after which the mice were euthanized, and medLNs and lungs were collected for analysis. Lymphocytes were isolated from the tissues as described (*83*). Briefly, the lungs were perfused with 1 ml of PBS, harvested, minced, and digested with collagenase (1 mg/ml, Sigma-Aldrich) and deoxyribonuclease (1 mg/ml, Sigma-Aldrich) in 3 ml of RPMI 1640 medium for 45 min at 37°C. Digested lungs and medLNs were passed through 40 μM cell strainer (BD Biosciences) to prepare single-cell suspension. Lymphocytes from lungs were obtained on a 44/67% Percoll gradient (Sigma-Aldrich) after centrifugation at 2000 rpm for 20 min at 4°C. To measure antigen-specific cytokine production, the cells were stimulated with 1 mM of influenza NP (311-325) peptide (#AS-62420, AnaSpec, Inc) and 1× of protein transport inhibitor (#00-4980-03, eBioscience) for 5 hours at 37°C. Antiviral T cell responses were evaluated by staining the cell suspensions with influenza PR8-specific NP311-325 (MHC class II) tetramer conjugated to BV421 (provided by NIH Tetramer Core Facility) for 30 min at 37°C followed by surface and intracellular staining and assessment by flow cytometry.

### Adoptive T_reg_ transfer in T cell–induced colitis mouse model

Naïve (CD4^+^CD25^−^CD45RB^hi^) T cells were sorted from the spleen and lymph nodes of CD45.1 congenic mice using a FACSAria cell sorter. Simultaneously, (CD4^+^Foxp3^+^) T_reg_ cells were sorted from the spleen and lymph nodes of CD45.2 WT (Foxp3^YFP-cre^) and CD45.2 CKO (*Prdm1*^fl/fl^Foxp3^YFP-cre^) mice, respectively. Colitis was induced by the intravenous injection of 5 × 10^5^ naïve CD4^+^ T cells into the *Rag2^−/−^* mice. To evaluate the in vivo T_reg_ suppressive activity in the presence or absence of BLIMP1, 1 × 10^5^ T_reg_ cells from WT or *Prdm1*-CKO mice were cotransferred intravenously with 5 × 10^5^ naïve CD4^+^ T cells into the *Rag2^−/−^* mice to monitor the development of colitis. The recipient mice were weighed weekly and euthanized 8 weeks after the cell transfer when the signs of colitis such as diarrhea appeared or when they lost more than 20% of their initial body weight. At the end of the experiment, mesLNs and spleen were examined for the protein expression levels by intracellular staining. Proximal, middle, and distal colon samples were excised, fixed in 10% formalin (#HT501128, Sigma-Aldrich), embedded in paraffin for cross-sectioning and stained with hematoxylin and eosin for histology imaging. The tissue section images for analysis were obtained using a Hamamatsu NanoZoomer slide scanner. Colon pathology scores were determined blindly on the basis of the criteria that includes the presence and number of crypt abscesses and submucosal inflammation (0 to 3), lamina propria cellularity/inflammatory infiltration (0 to 3), epithelial hyperplasia and goblet cell depletion (0 to 3), and the percentage of tissue involvement (0 to 3); where 0 = normal, 1 = mild, 2 = moderate, and 3 = severe (*74*, *80*, *84*).

### RNA-seq and analysis

Purified CD4^+^ T cells from medLNs of influenza-infected WT (*Prdm1*^fl/fl^) and *Prdm1* CKO (*Prdm1*^fl/fl^CD4^cre^) mice were rested overnight in complete medium and restimulated with recombinant human IL-2 (200 IU/ml) for 24 hours at 37°C. Cells were harvested and total RNA was extracted using Direct-zol RNA MiniPrep kit (#R2052, Zymo Research) according to the kit’s protocol. Two hundred nanograms of total RNA was used to prepare RNA-seq libraries using the KAPA RNA HyperPrep Kit (#KK8542, KAPABIOSYSTEMS) per the manufacturer’s protocol. The libraries were barcoded (indexed) and sequenced on an Illumina NovaSeq platform. Sequenced reads (50 bp, single end) were obtained with the Illumina CASAVA pipeline and mapped to the mouse genome (mm10/GRCm38) using Bowtie 2.2.6 (*85*) and TopHat 2.2.1 (*86*). Raw counts that fell on exons of each gene were calculated and normalized by using RPKM (reads per kilobase per million mapped reads). DEGs were identified using the R Bioconductor package “edgeR” (*87*), using thresholds FDR < 0.05 and |log_2_FC| > =1, and expression heatmaps were generated with the R package “pheatmap” (*88*). For gene set enrichment analysis, RNA-seq–based gene expression data were compared with molecular signature gene sets using R package “fgsea” (*89*).

Splenic T_reg_ cells from WT (Foxp3^YFP-cre^) and *Prdm1*-CKO (*Prdm1*^fl/fl^Foxp3^YFP-cre^) mice were isolated, preactivated with anti-CD3 + anti-CD28, rested overnight, and cultured with IL-2 for 24 hours. Cells were harvested, total RNA was extracted, and 100 ng of total RNA was used to prepare RNA-seq libraries as above. Fifty-bp PE reads were produced on an Illumina NovaSeq platform. RNA-seq data were processed using the RNA-seek workflow v1.8.0 (https://doi.org/10.5281/zenodo.5223025) and the NIH HPC Biowulf cluster. (http://hpc.nih.gov). In brief, the reads were trimmed using cutadapt v1.18 (*90*) and aligned to the mm10 reference genome and GENCODE release M21 using STAR v2.7.6a (*91*) in two-pass basic mode. Expression levels were quantified with RSEM v1.3.0 (*92*). Differential analysis was completed with limma 3.52.4 (*93*) with the following prefiltering parameters: minimum CPM of 0.5 in two libraries. Genes were considered significant if they had an FDR less than 0.05 and an absolute fold change greater than 2. Pathway analysis was assessed on differential genes using ClusterProfiler 4.4.4 (*94*) on the GO biological processes database from GO.db 3.15.0 (*95*) and the Hallmark mouse database from R package msigdbr 7.5.1 (*96*). Related significant GO pathways were collapsed using the simplify function. The resulting figures were created using the enrichplot package 1.16.2. Heatmaps of the FPKM-normalized data were created with ComplexHeatmap 2.12.1 (*97*).

### ChIP-seq and analysis

Purified human natural T_reg_ cells (CD4^+^CD25^+^CD127^low^) from HDs were cultured and expanded using T_reg_ expansion kit, human (#130-095-345, Miltenyi Biotec) in the presence of recombinant human IL-2 (500 IU/ml) according to the manufacturer’s instructions. ChIP-seq was performed as described earlier (*98*). Briefly, the cells were fixed in 1% formaldehyde for 10 min and sonicated to prepare chromatin followed by immunoprecipitation with rabbit anti-IgG (#2729S, Cell Signaling Technology) and two different antibodies for BLIMP1: anti-BLIMP1 Ab1 (#9115S, Cell Signaling Technology) and anti-BLIMP1 Ab2 (#MA5-14879, Thermo Fisher Scientific). DNA was end-repaired using an End-It DNA-Repair kit (Epicentre), and was indexed, amplified, and sequenced on an Illumina HiSeq-2500.

BLIMP1 ChIP-seq raw reads were processed into BEDPE and bigWig files as described previously (*99*). Replicates were combined for peak calling using cLoops2’s callPeaks module (*100*) with parameters -eps 150 and -minPts 10 against the IgG control. Only overlapped peaks from both donors and both BLIMP1 antibodies are used for the following analysis. Peak targets and distance to nearest gene transcription start sites were obtained with anoPeaks.py script from the cLoops2 package, and target genes GO terms enrichment analysis was performed by findGO.pl in the HOMER package (*101*). We considered terms with at least ten associated target genes and excluded terms containing more than 1000 genes, consistent with previous studies (*99*, *102*). Motif analysis was performed using findMotifsGenome.pl in the HOMER package. Genome-browser–like images were presented by the cLoops2 plot module.

### Ex vivo culture of CD4^+^ T cells from patients with ATL

Frozen PBMCs from HD and patients with acute ATL were thawed and rested overnight in complete TexMACS medium supplemented with 5% human AB serum, 2 mM l-glutamine, and penicillin (50 U/ml) + streptomycin (50 μg/ml) at 37°C. CD4^+^ T cells were isolated from the PBMCs and stimulated using plate-bound anti-CD3 (2 μg/ml) and soluble anti-CD28 (1 μg/ml) and cultured in complete medium in the presence of recombinant human IL-2 (500 IU/ml) for 24 hours at 37°C, after which the cells were harvested for the protein expression analysis by flow cytometry.

### Lentiviral transduction in ATL cells

Human *PRDM1* cDNA was PCR-amplified from mRNA extracted from purified human CD8^+^ T cell using the following primers: forward (5′-ctagGAATTCatgttggatatttgcttg-3′) and reverse (5′-gattGCGGCCGCttaaggatccattggttc-3′). The PCR product was digested with restriction enzymes EcoRI and NotI, purified, and ligated into the pLVX-EF1a-IRES-ZsGreen1 lentiviral vector. The ligation mixture was transformed into *Escherichia coli* DH5α cells, and positive clones were selected. Recombinant plasmids were confirmed by sequencing to verify correct insertion and orientation of the *PRDM1* cDNA. Lentiviruses were generated by transfecting the *PRDM1* plasmids with the pCMV-VSV-G (#8454, Addgene) and pCMV-dR8.2 dvpr (#8455, Addgene) packaging plasmids into 293T cells. Lentiviral supernatants carrying the pLVX-EV (empty vector) or pLVX-*PRDM1* plasmids were mixed with polybrene (8 μg/ml; #TR-1003, Sigma-Aldrich) and transduced into preactivated mouse CD4^+^ T cells from frozen PBMCs of patients with ATL by centrifugation at 3500 rpm for 45 min at 30°C. The lentiviral supernatants were replaced with new medium containing human recombinant IL-2 (100 IU/ml), and the cells were cultured for 2 days at 37°C and harvested for further analysis.

### scRNA-seq and analysis

Purified CD4^+^ T cells from frozen PBMCs of HDs and patients with acute ATL were stimulated briefly with plate-bound anti-CD3 (2 μg/ml) and soluble anti-CD28 (1 μg/ml) in the presence of recombinant human IL-2 (500 IU/ml) for 4 hours at 37°C. The cells were harvested and loaded into separate channels of a single-cell Chip G with reverse transcriptase reagent mixture and 5′ gel beads according to the manufacturer’s protocol (10X Genomics; Pleasanton, CA). The chips were next loaded into the 10X Genomics Chromium Controller for single-cell partitioning, immediately followed by emulsion recovery from the chip and incubated in an MJ Research PTC-200 Thermal Cycler for the reverse transcription reaction; cDNA isolation and library preparation were completed per the manufacturer’s protocol. Isolated cDNA was amplified (11 cycles) and was used for the preparation of a gene expression library with the Chromium Next GEM Single Cell 3’ Reagent kits v3.1 (10X Genomics). The quality of the cDNA and library was evaluated using the D1000 high-sensitivity kits on a 2200 TapeStation system (Agilent, Santa Clara, CA). The gene expression libraries were sequenced on the Illumina HiSeq platform. Sequencing data were processed with 10X Genomics Cell Ranger version 7.2.0 (*103*). Cell Ranger “mkfastq” function was used to generate fastqs and “count” was used to generate gene expression data using the Human reference (GRCh38) - 2020-A transcriptome reference. Analyses were primarily done in Seurat version 5.0.1 (*104*). Cells containing <500 unique molecular identifiers (UMIs) and > 10% mitochondrial gene percentage were filtered out. Features found in <3 cells were also removed. Doublets were identified using the scDblFinder R package version 1.16 (*105*) and were also filtered out. *PRDM1^+^* cells were defined as having any detectable expression of *PRDM1*. Data from each individual sample were normalized and scaled. Dimensionality reduction (dims = 1:20) was performed with “RunPCA” and “RunUMAP” functions in Seurat. Shared nearest-neighbor graphs–based clustering was performed using the Louvain algorithm (resolution = 0.2) with “FindClusters” function in Seurat. All individual samples were integrated together using Seurat’s “FindIntegrationAnchors” method with “rpca” reduction and k.weight set to 70. The integrated data were then reprocessed with Seurat as described above—normalizing, scaling, dimensionality reduction, and clustering. Cell type annotation was performed with Azimuth R package version 0.4.6 using the provided human PBMC reference (*106*). Cells annotated as “CD4 T” in the level 1 cell type annotation from Azimuth were used for downstream analyses. Cells with the level 2 cell type annotation as CD4 naïve (*n* = 321), CD4 proliferating (*n* = 65), and CD4 effector memory (*n* = 18) were merged into the “Other” category as these cells were present in fewer numbers. Differential expression analyses were performed using the Wilcoxon ranked sum test with the “FindMarkers” function in Seurat for the following groups of cell populations: ATL versus HD in all cells and *PRDM1*^+^ versus *PRDM1*^−^ in T_reg_ cells. Pathway enrichment analyses were conducted using Qiagen IPA software (QIAGEN Inc., https://digitalinsights.qiagen.com/IPA) version 107193442 (*107*). The log_2_FC and *P* value from each differential expression comparison was used as input to the IPA software. A *P* value cutoff of 0.05 was used in the IPA software before running pathway enrichment analysis. Pathways from IPA were subset to just those with a −log(*P* value) of >6 and ratio of >0.5, where the ratio is the number of genes in the differential expression results that are also in the pathway divided by the total genes in the pathway.

### Quantification and statistical analysis

Figures 1A, 2A, 3A, 3F, 4G, 4J, 6A, 6L, and 7A and fig. S1A were created with BioRender.com. GraphPad Prism 10 (La Jolla, CA, USA) was used for statistical analysis. Two-tailed Student’s *t* test for comparison of means between two groups, and one-way analysis of variance (ANOVA) for comparison of means between more than two groups were used. All statistical tests were followed by Tukey’s multiple comparison’s posttest. Differences were considered statistically significant if the *P* value was <0.05. The data depicted in the bar graphs and scatter dot plots are represented as means ± SEM.

## Supplementary Materials

### The PDF file includes:

Figs. S1 to S7 Legends for tables S1 to S4 Tables S5 to S8 References
