# Supplementary material for: BLIMP1 negatively regulates IL-2 signaling in T cells
Source: Sci Adv. 2025 Jul 18;11(29):eadx8105. doi: 10.1126/sciadv.adx8105 (PMC12273773; doi:10.1126/sciadv.adx8105)
Supplement: Supplementary file 1 — Figs. S1 to S7 Legends for tables S1 to S4 Tables S5 to S8 References [file sciadv.adx8105_sm.pdf]

Science Advances
AAAS

Supplementary Materials for

BLIMP1 negatively regulates IL-2 signaling in T cells

Suyasha Roy *et al.*

Corresponding author: Warren J. Leonard, leonardw@nhlbi.nih.gov

Sci. Adv. **11**, eadx8105 (2025)
DOI: 10.1126/sciadv.adx8105

The PDF file includes:

Figs. S1 to S7
Legends for tables S1 to S4
Tables S5 to S8
References

Other Supplementary Material for this manuscript includes the following:

Tables S1 to S4

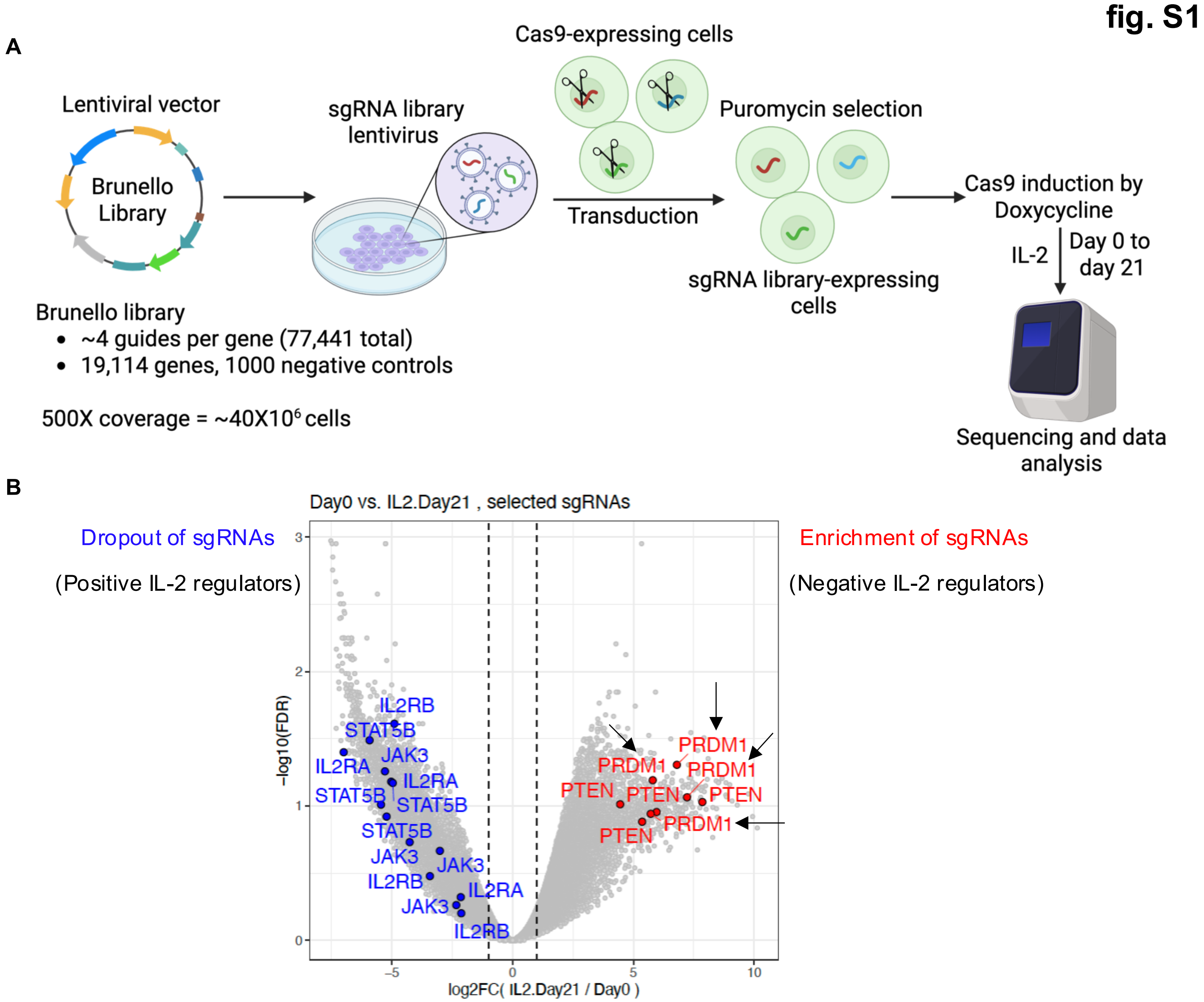

Supplementary Fig. S1: CRISPR/Cas9 knockout screen in IL-2-dependent ED40515(+) cells. (**A**) Schematic representation of the experimental protocol for the CRISPR/Cas9 knockout screening. Cells were transduced with the lentiviral plasmid carrying Brunello sgRNAs library followed by puromycin selection. Cas9 expression was induced with doxycycline, and the cells were then cultured for 21 days in the presence of IL-2. Created in BioRender. Roy, S. (2025) https://BioRender.com/g2okxin. (**B**) Volcano plot showing enrichment (positive selection) and dropout (negative selection) of sgRNAs after 21 days of IL-2 treatment relative to day 0.

Fig. S2

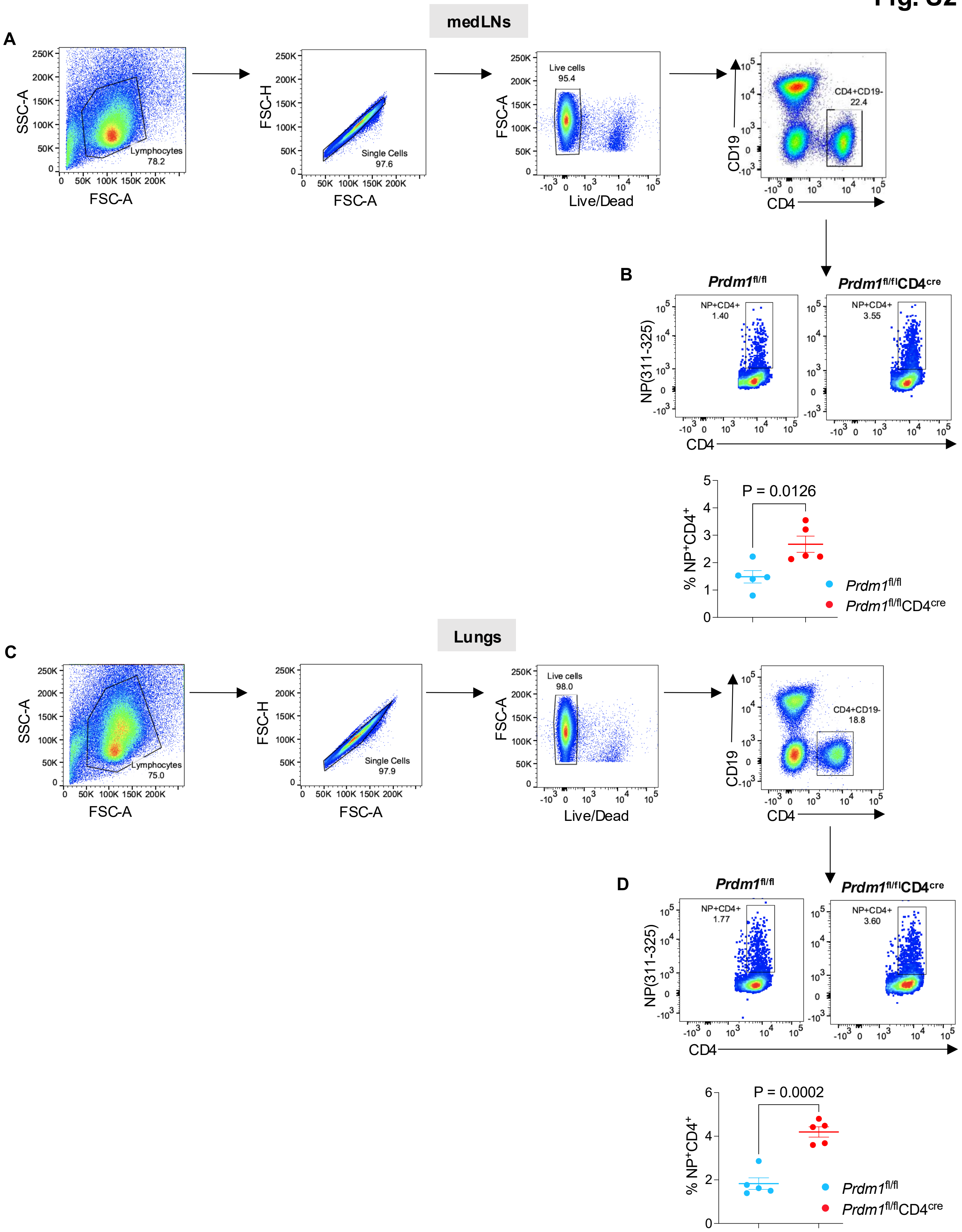

medLNs
A
SSC-A
FSC-A
Lymphocytes
78.2
FSC-H
Single Cells
97.6
Live cells
95.4
Live/Dead
CD4+CD19-
22.4
CD19
CD4
B
Prdm1fl/fl
Prdm1fl/flCD4cre
NP+CD4+
1.40
NP+CD4+
3.55
NP(311-325)
P = 0.0126
% NP+CD4+
Lungs
C
Lymphocytes
75.0
Single Cells
97.9
Live cells
98.0
CD4+CD19-
18.8
D
NP+CD4+
1.77
NP+CD4+
3.60
P = 0.0002

Supplementary Fig. S2: Increased frequency of influenza infected CD4^{+} T cells in *Prdm1*-CKO mice. (**A** to **D**) *Prdm1*$^{fl/fl}$ (WT) and *Prdm1*$^{fl/fl}$CD4^{cre} (*Prdm1*-CKO) mice were infected intranasally with 1000 VFU of PR8 influenza virus. At day 10, medLNs and lungs were harvested, and single cell suspensions were prepared. Cells were stimulated with NP$_{311\text{-}325}$ peptide (1 μM) for 5 hours at 37°C, stained and subjected to flow cytometric analysis. (A) Gating strategy for NP$_{311\text{-}325}$ tetramer^{+} influenza-specific CD4^{+} T cells in the medLNs. (B) Frequency of NP$_{311\text{-}325}$ tetramer^{+} influenza-specific CD4^{+} T cells in the medLNs from *Prdm1*$^{fl/fl}$ and *Prdm1*$^{fl/fl}$CD4^{cre} mice. Data are representative of mean ± SEM from two independent experiments (n = 5 mice/group). Two-tailed unpaired Student's t test was used for statistical analysis. (C) Gating strategy for NP$_{311\text{-}325}$ tetramer^{+} influenza-specific CD4^{+} T cells in the lungs. (D) Frequency of NP$_{311\text{-}325}$ tetramer^{+} influenza-specific CD4^{+} T cells in the lungs of *Prdm1*$^{fl/fl}$ and *Prdm1*$^{fl/fl}$CD4^{cre} mice. Data are representative of mean ± SEM from two independent experiments (n = 5 mice/group). Two-tailed unpaired Student's t test was used for statistical analysis.

Fig. S3

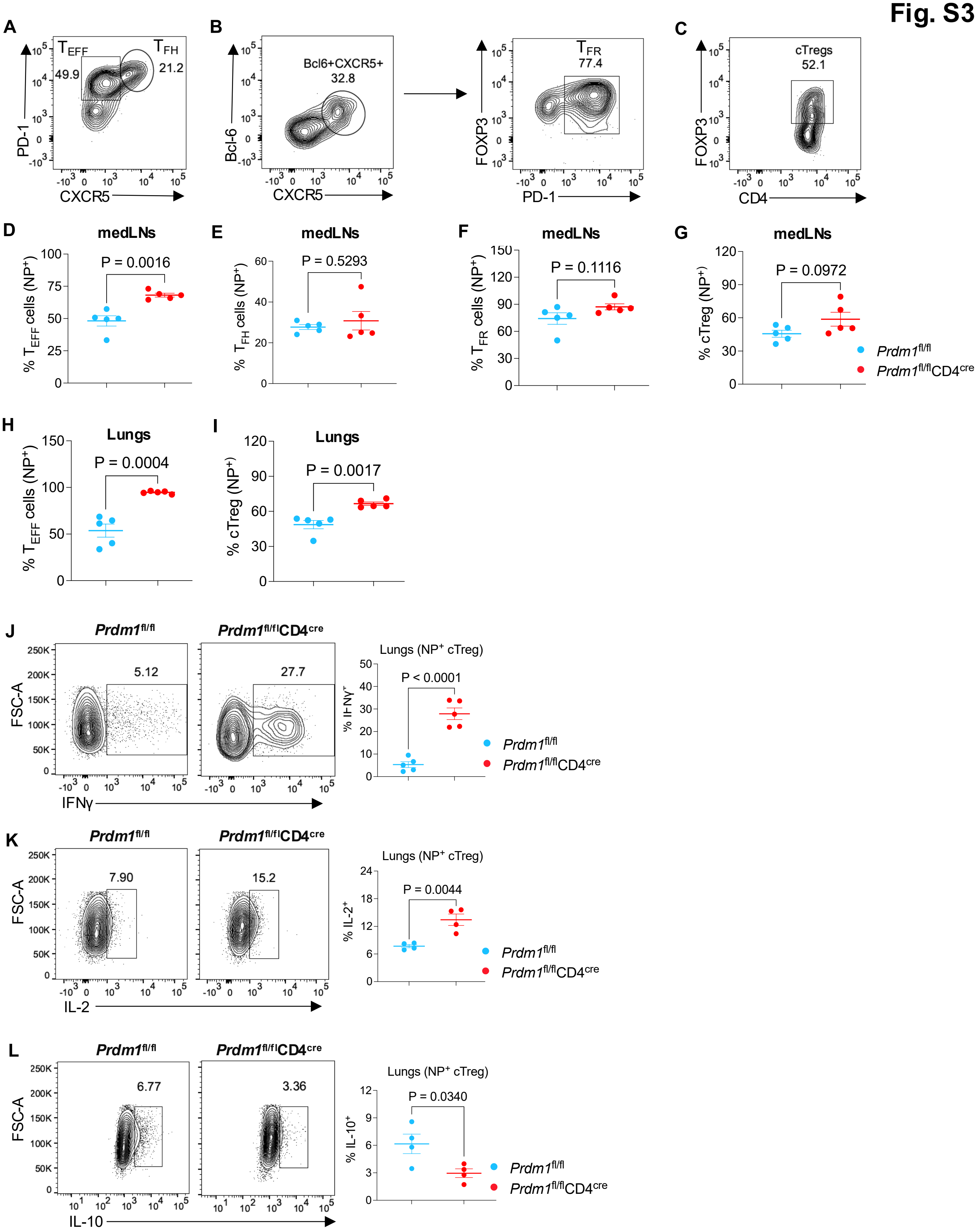

A
T_{EFF}
49.9
T_{FH}
21.2
PD-1
CXCR5
B
Bcl6+CXCR5+
32.8
Bcl-6
CXCR5
T_{FR}
77.4
FOXP3
PD-1
C
cTregs
52.1
FOXP3
CD4
D
medLNs
P = 0.0016
% T_{EFF} cells (NP+)
E
medLNs
P = 0.5293
% T_{FH} cells (NP+)
F
medLNs
P = 0.1116
% T_{FR} cells (NP+)
G
medLNs
P = 0.0972
% cTreg (NP+)
Prdm1fl/fl
Prdm1fl/flCD4cre
H
Lungs
P = 0.0004
% T_{EFF} cells (NP+)
I
Lungs
P = 0.0017
% cTreg (NP+)
J
Prdm1fl/fl
Prdm1fl/flCD4cre
5.12
27.7
FSC-A
IFNγ
Lungs (NP+ cTreg)
P < 0.0001
% IFNγ+
Prdm1fl/fl
Prdm1fl/flCD4cre
K
Prdm1fl/fl
Prdm1fl/flCD4cre
7.90
15.2
FSC-A
IL-2
Lungs (NP+ cTreg)
P = 0.0044
% IL-2+
Prdm1fl/fl
Prdm1fl/flCD4cre
L
Prdm1fl/fl
Prdm1fl/flCD4cre
6.77
3.36
FSC-A
IL-10
Lungs (NP+ cTreg)
P = 0.0340
% IL-10+
Prdm1fl/fl
Prdm1fl/flCD4cre

Supplementary Fig. S3: Altered immune response in influenza virus-infected *Prdm1*-CKO mice. (**A** to **L**) *Prdm1*$^{fl/fl}$ (WT) and *Prdm1*$^{fl/fl}$CD4^{cre} (*Prdm1*-CKO) mice were infected intranasally with 1000 VFU of PR8 influenza virus. At day 10, medLNs and lungs were harvested, and single cell suspensions were prepared. Cells were stimulated with $NP_{311-325}$ peptide (1 μM) for 5 hours at 37°C, stained and subjected to flow cytometric analysis. (A) Gating strategy for $NP_{311-325}$ tetramer$^+$ influenza-specific PD1$^+$CXCR5$^+$ T_{FH} and PD1$^+$CXCR5$^-$ T_{EFF} cells. (B) Gating strategy for $NP_{311-325}$ tetramer$^+$ influenza-specific Bcl-6$^+$CXCR5$^+$ PD1$^+$Foxp3$^+$ T_{FR} cells. (C) Gating strategy for $NP_{311-325}$ tetramer$^+$ influenza-specific CD4$^+$Foxp3$^+$ cTregs. (D to G) Flow cytometric analysis of frequency of $NP_{311-325}$ tetramer$^+$ influenza-specific (D) T_{EFF}, (E) T_{FH}, (F) T_{FR} and (G) cTregs in the medLNs from *Prdm1*$^{fl/fl}$ and *Prdm1*$^{fl/fl}$CD4^{cre} mice. (H and I) Flow cytometry analysis of frequency of $NP_{311-325}$ tetramer$^+$ influenza-specific (H) T_{EFF} and (I) cTregs in the lungs from *Prdm1*$^{fl/fl}$ and *Prdm1*$^{fl/fl}$CD4^{cre} mice. (J to L) Flow cytometry analysis of (J) IFNγ, (K) IL-2 and (L) IL-10 production by influenza-specific cTregs in the lungs of *Prdm1*$^{fl/fl}$ and *Prdm1*$^{fl/fl}$CD4^{cre} mice. Data are representative of mean ± SEM from two independent experiments (n = 4-5 mice/group). Two-tailed unpaired Student's t test was used for statistical analysis.

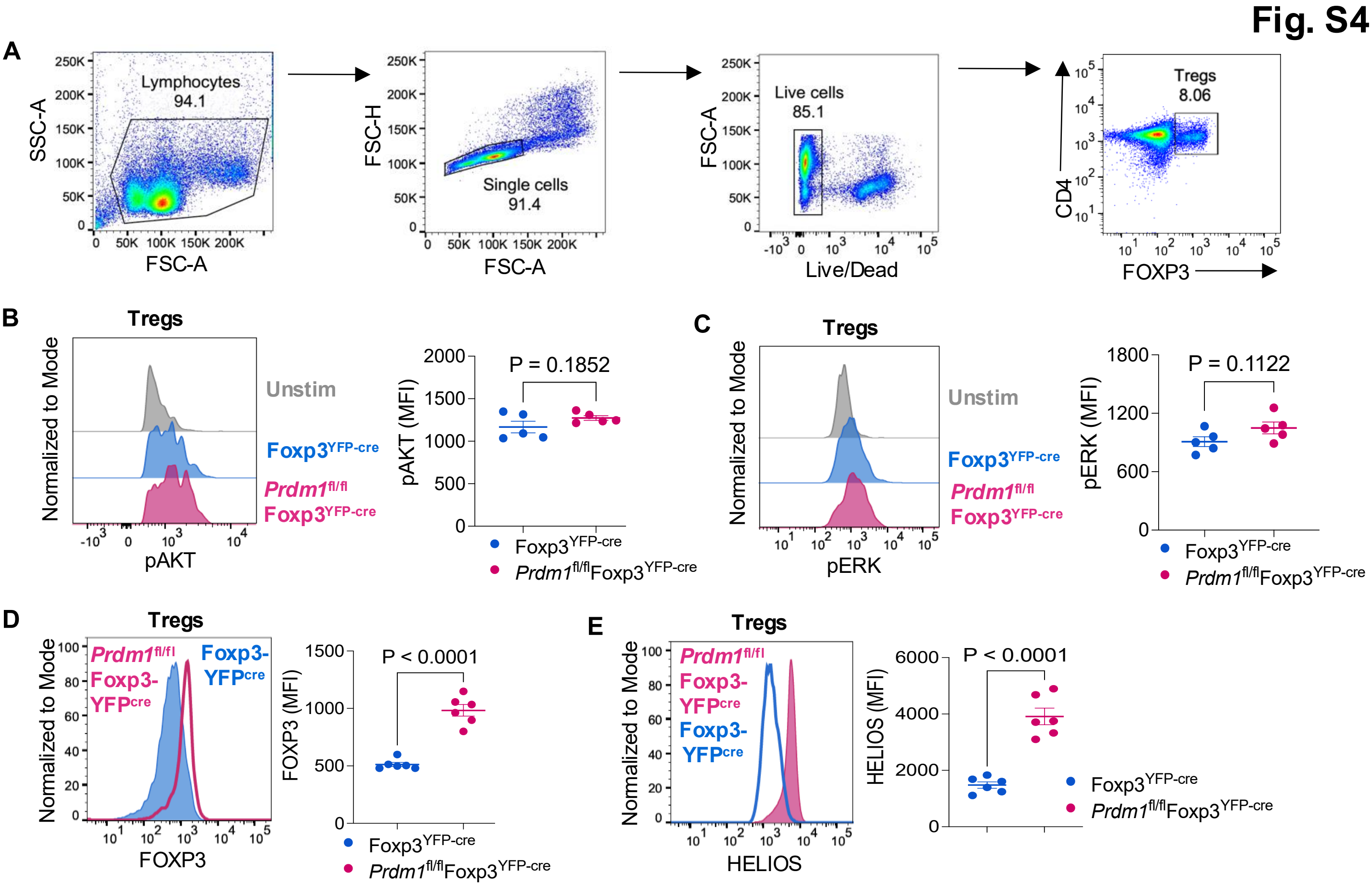

Supplementary Fig. S4: BLIMP1 regulates signature markers in mouse Tregs. (**A** to **E**) Tregs were purified from the spleens of Foxp3$^{YFP\text{-}cre}$ (WT) and *Prdm1*$^{fl/fl}$Foxp3$^{YFP\text{-}cre}$ (CKO) mice and stimulated with anti-CD3 and anti-CD28 in the presence of 500 IU/ml of IL-2 for 72 hours at 37°C. Cells were harvested and stained, and live cells were gated as CD4^{+}Foxp3^{+} Tregs based on YFP expression as assessed by flow cytometry. (A) Gating strategy for the experiment. (B to E) Plots with statistical representation for protein expression of (B) pAKT, (C) pERK, (D) FOXP3 and (E) HELIOS. Data are representative of mean ± SEM from three independent experiments (n = 5-6 individual mice/group). Two-tailed unpaired Student's t test was used for statistical analysis.

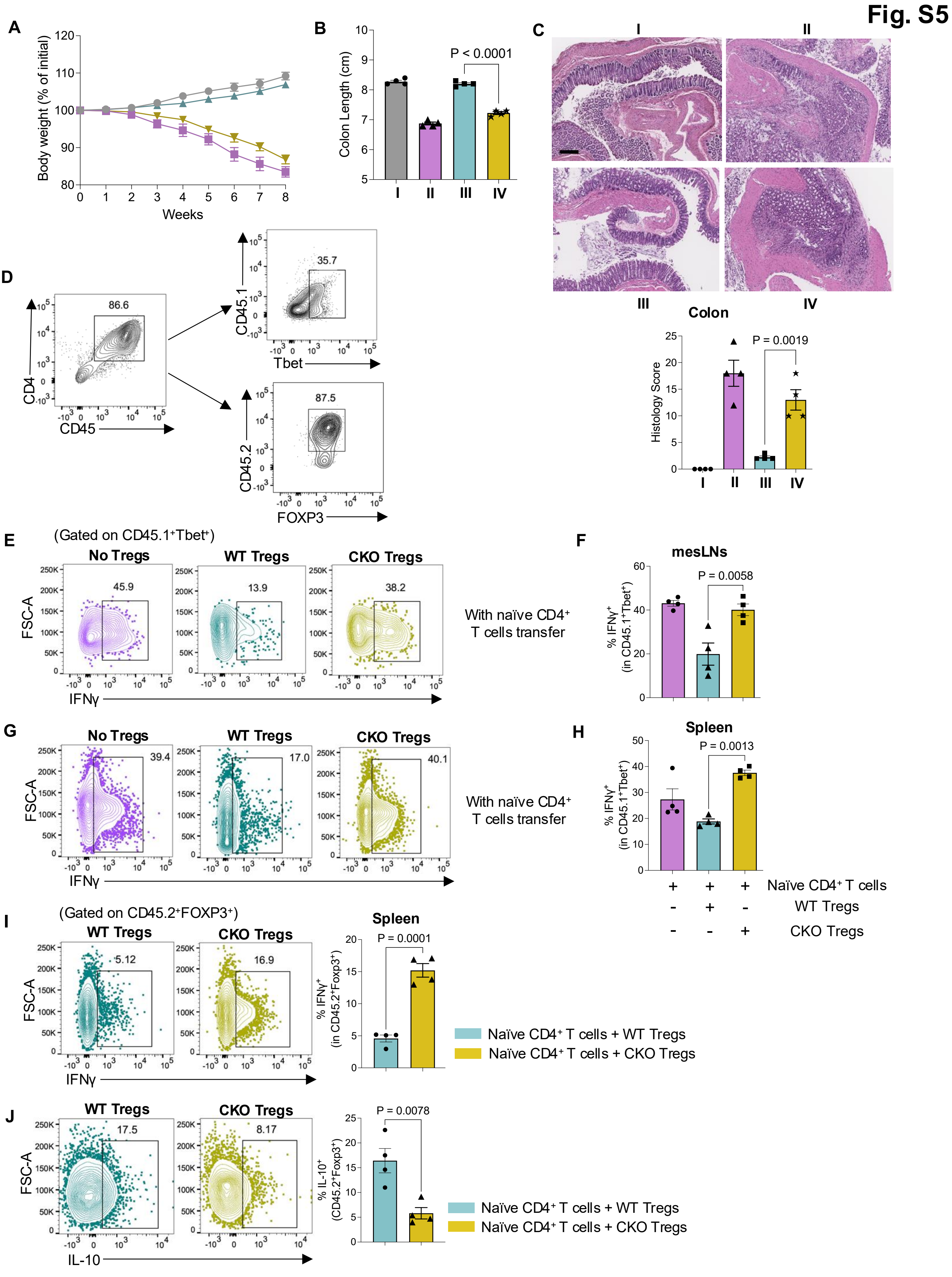
Fig. S5
A
Body weight (% of initial)
Weeks
B
Colon Length (cm)
P < 0.0001
I
II
III
IV
C
Colon
Histology Score
P = 0.0019
D
86.6
CD4
CD45
35.7
CD45.1
Tbet
87.5
CD45.2
FOXP3
E
(Gated on CD45.1⁺Tbet⁺)
No Tregs
WT Tregs
CKO Tregs
45.9
13.9
38.2
FSC-A
IFNγ
With naïve CD4⁺ T cells transfer
F
mesLNs
P = 0.0058
% IFNγ⁺ (in CD45.1⁺Tbet⁺)
G
No Tregs
WT Tregs
CKO Tregs
39.4
17.0
40.1
FSC-A
IFNγ
With naïve CD4⁺ T cells transfer
H
Spleen
P = 0.0013
% IFNγ⁺ (in CD45.1⁺Tbet⁺)
Naïve CD4⁺ T cells
WT Tregs
CKO Tregs
I
(Gated on CD45.2⁺FOXP3⁺)
WT Tregs
CKO Tregs
5.12
16.9
FSC-A
IFNγ
Spleen
P = 0.0001
% IFNγ⁺ (in CD45.2⁺Foxp3⁺)
Naïve CD4⁺ T cells + WT Tregs
Naïve CD4⁺ T cells + CKO Tregs
J
WT Tregs
CKO Tregs
17.5
8.17
FSC-A
IL-10
P = 0.0078
% IL-10⁺ (CD45.2⁺Foxp3⁺)
Naïve CD4⁺ T cells + WT Tregs
Naïve CD4⁺ T cells + CKO Tregs

Supplementary Fig. S5: *Prdm1*-CKO impairs the suppressive function of mouse Tregs. Adoptive Treg transfer from Foxp3$^{YFP\text{-}cre}$ (WT) or *Prdm1*$^{fl/fl}$Foxp3$^{YFP\text{-}cre}$ (CKO) mice to *Rag2*$^{-/-}$ mice with naïve CD4^{+} T-cell transfer induced colitis. (**A**) Changes in the body weight over 8 weeks after the T-cell transfer. (**B**) Differences in the length of the colon (in cm) at the end of the study after 8 weeks post T-cells transfer. (**C**) Representative colon histology images with H&E staining for groups I, II, III and IV. The bar indicates 200 mm, with colonic histology scores at the endpoint of the study (8 weeks) following the T-cell transfer. (**D**) Gating strategy for CD45.1^{+}Tbet^{+} and CD45.2^{+}FOXP3^{+} T cells. **(E-J)** Flow cytometry analysis of CD45.1^{+}Tbet^{+} IFNγ^{+} T cells in the (E and F) mesLNs and (G and H) spleen. Plots are representative of mean ± SEM from two independent experiments (n = 4 mice/group) using one-way ANOVA for the statistical analysis. (I and J) Flow cytometry analysis for (I) IFNγ and (J) IL-10 expression in CD45.2^{+}FOXP3^{+} Tregs in the spleen. Plots are representative of mean ± SEM from two independent experiments (n = 4 mice/group) using two-tailed unpaired Student's t test for the statistical analysis.

Fig. S6

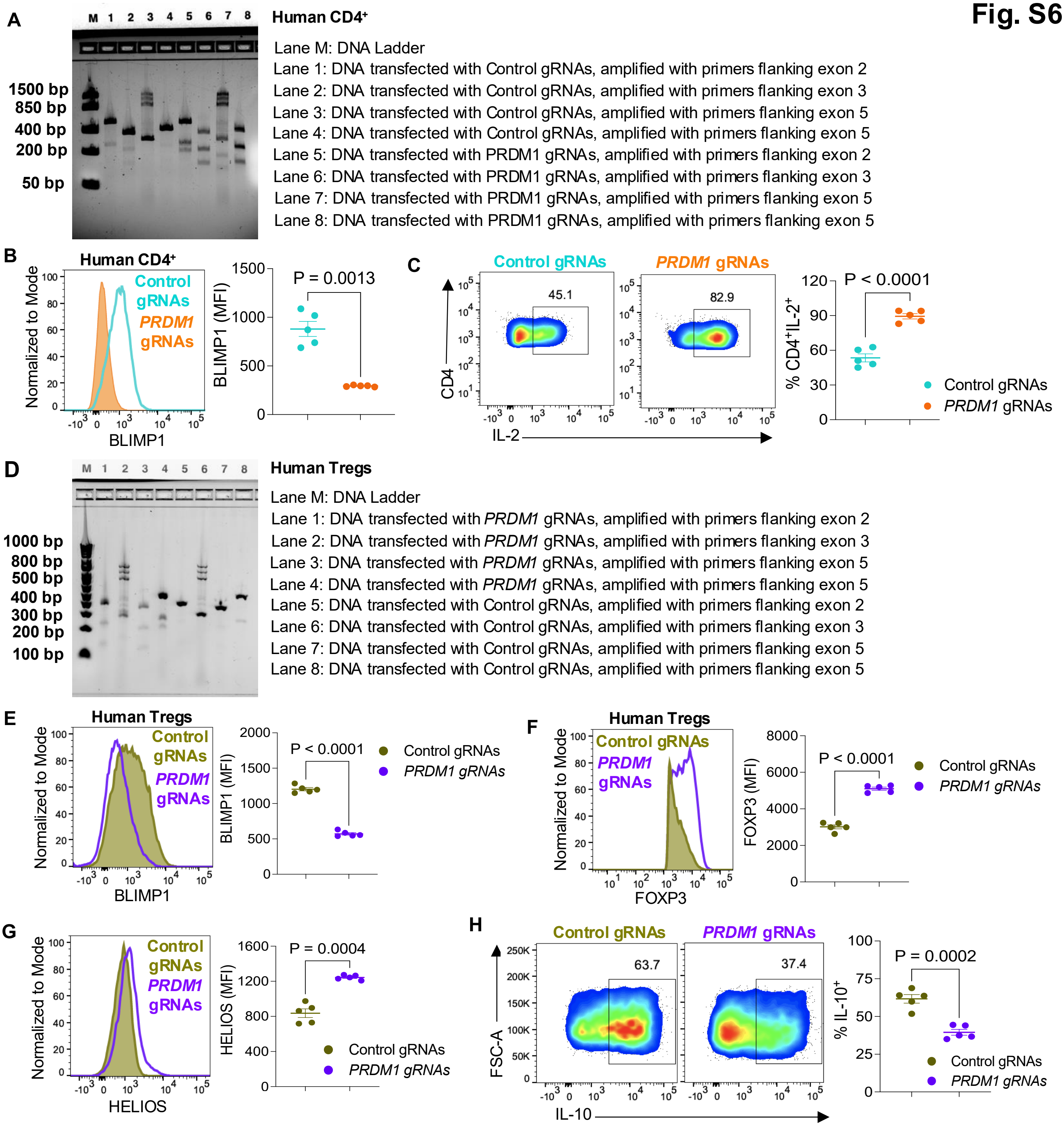
A
M 1 2 3 4 5 6 7 8
1500 bp
850 bp
400 bp
200 bp
50 bp
Human CD4+
Lane M: DNA Ladder
Lane 1: DNA transfected with Control gRNAs, amplified with primers flanking exon 2
Lane 2: DNA transfected with Control gRNAs, amplified with primers flanking exon 3
Lane 3: DNA transfected with Control gRNAs, amplified with primers flanking exon 5
Lane 4: DNA transfected with Control gRNAs, amplified with primers flanking exon 5
Lane 5: DNA transfected with PRDM1 gRNAs, amplified with primers flanking exon 2
Lane 6: DNA transfected with PRDM1 gRNAs, amplified with primers flanking exon 3
Lane 7: DNA transfected with PRDM1 gRNAs, amplified with primers flanking exon 5
Lane 8: DNA transfected with PRDM1 gRNAs, amplified with primers flanking exon 5
B
Human CD4+
Control gRNAs
PRDM1 gRNAs
Normalized to Mode
BLIMP1
BLIMP1 (MFI)
P = 0.0013
C
Control gRNAs
PRDM1 gRNAs
45.1
82.9
CD4
IL-2
% CD4+IL-2+
P < 0.0001
Control gRNAs
PRDM1 gRNAs
D
M 1 2 3 4 5 6 7 8
1000 bp
800 bp
500 bp
400 bp
300 bp
200 bp
100 bp
Human Tregs
Lane M: DNA Ladder
Lane 1: DNA transfected with PRDM1 gRNAs, amplified with primers flanking exon 2
Lane 2: DNA transfected with PRDM1 gRNAs, amplified with primers flanking exon 3
Lane 3: DNA transfected with PRDM1 gRNAs, amplified with primers flanking exon 5
Lane 4: DNA transfected with PRDM1 gRNAs, amplified with primers flanking exon 5
Lane 5: DNA transfected with Control gRNAs, amplified with primers flanking exon 2
Lane 6: DNA transfected with Control gRNAs, amplified with primers flanking exon 3
Lane 7: DNA transfected with Control gRNAs, amplified with primers flanking exon 5
Lane 8: DNA transfected with Control gRNAs, amplified with primers flanking exon 5
E
Human Tregs
Control gRNAs
PRDM1 gRNAs
Normalized to Mode
BLIMP1
BLIMP1 (MFI)
P < 0.0001
F
Human Tregs
Control gRNAs
PRDM1 gRNAs
Normalized to Mode
FOXP3
FOXP3 (MFI)
P < 0.0001
G
Control gRNAs
PRDM1 gRNAs
Normalized to Mode
HELIOS
HELIOS (MFI)
P = 0.0004
H
Control gRNAs
PRDM1 gRNAs
63.7
37.4
FSC-A
IL-10
% IL-10+
P = 0.0002

Supplementary Fig. S6: CRISPR/Cas9-mediated *PRDM1* deletion in human CD4$^+$ T cells and natural Tregs. (**A** to **C**) Human CD4$^+$ T cells were purified from the buffy coats from healthy individuals and preactivated with anti-CD3 and anti-CD28 for 48 hours at 37°C. Cells were electroporated with control gRNAs or *PRDM1* gRNAs annealed with Cas9 as a RNP complex. Cells were then cultured with 200 IU/ml of IL-2 for 72 hours at 37°C. Cells were harvested and stained for flow cytometry analysis. (A) T7 endonuclease I assay showing *PRDM1* deletion at the DNA level in human CD4$^+$ T cells by *PRDM1* gRNAs, with one gRNA for targeting exon 2, one for exon 3, and two for exon 5. (B) Histograms with statistical representation showing BLIMP1 staining by flow cytometry. (C) Flow cytometry analysis of IL-2 production with control versus *PRDM1* gRNAs. Data are representative of mean ± SEM from three independent experiments (n = 5 individuals). Two-tailed paired Student's t test was used for statistical analysis. (**D** to **H**) Human CD4$^+$CD25$^+$CD127^{low} natural Tregs were purified from the buffy coats of healthy individuals and stimulated with anti-CD3 and anti-CD28 in the presence of 500 IU/ml IL-2 and expanded for 14 days at 37°C. Cells were then electroporated with control gRNAs or *PRDM1* gRNAs annealed with Cas9 as an RNP complex, and cultured with 500 IU/ml IL-2 for 72 hours at 37°C. Cells were then harvested and stained for flow cytometry analysis. (D) T7 endonuclease I assay showing *PRDM1* deletion at the DNA level in human Tregs by four different *PRDM1* gRNAs, with one gRNA for targeting exon 2, one for exon 3, and two for exon 5. (E to G) Histograms with statistical representation showing (E) BLIMP1, (F) FOXP3 and (G) HELIOS staining by flow cytometry. (H) Flow cytometric analysis of the percentage of IL-10-producing Tregs with control versus *PRDM1* gRNAs. Data are representative of mean ± SEM from three independent experiments (n = 5 individuals per group). Two-tailed paired Student's t test was used for statistical analysis.

Fig. S7

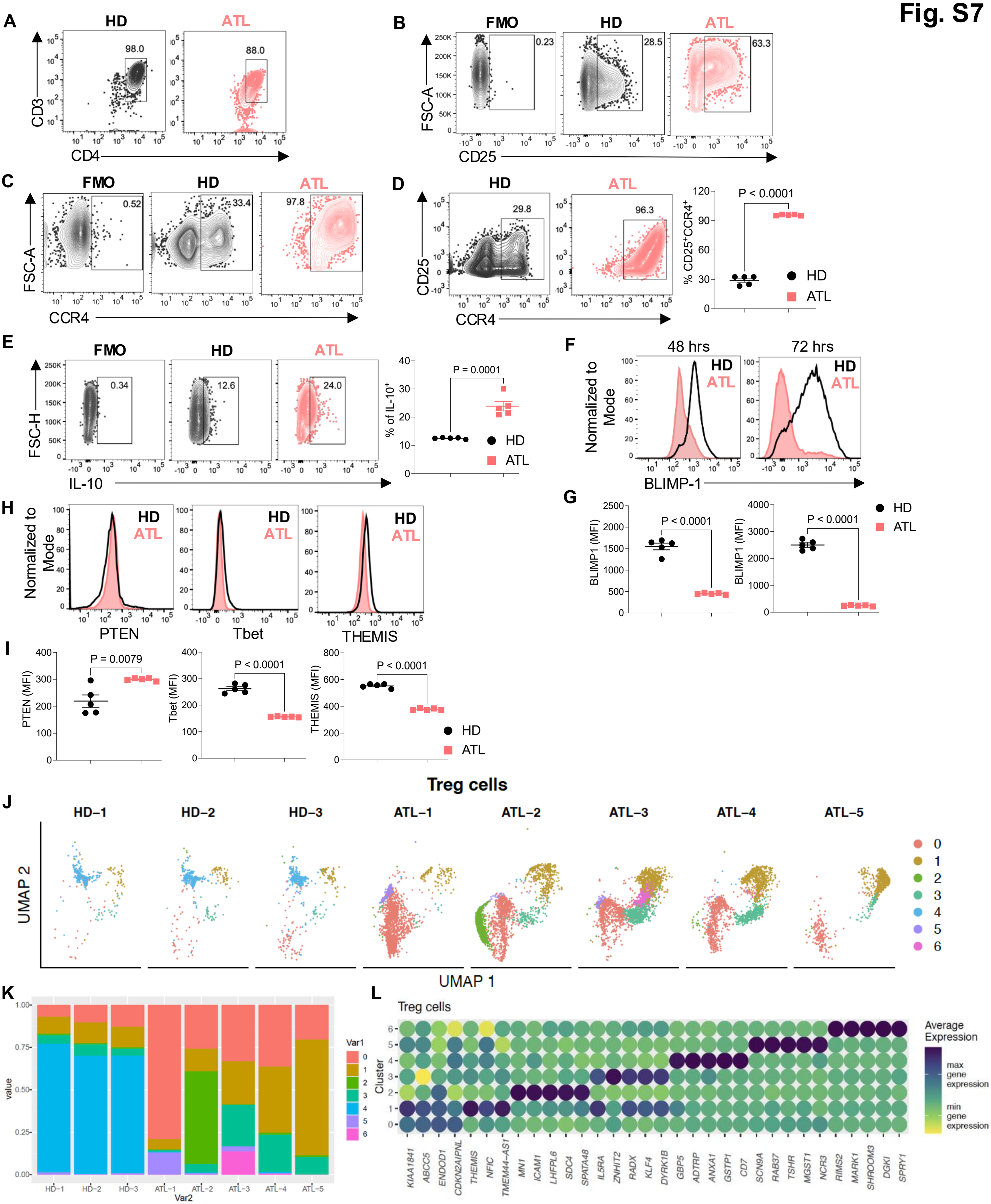

A
HD
ATL
98.0
88.0
CD3
CD4
B
FMO
HD
ATL
0.23
28.5
63.3
FSC-A
CD25
C
FMO
HD
ATL
0.52
33.4
97.8
FSC-A
CCR4
D
HD
ATL
29.8
96.3
CD25
CCR4
P < 0.0001
% CD25+CCR4+
HD
ATL
E
FMO
HD
ATL
0.34
12.6
24.0
FSC-H
IL-10
P = 0.0001
% of IL-10+
HD
ATL
F
48 hrs
72 hrs
HD
ATL
Normalized to Mode
BLIMP-1
G
P < 0.0001
P < 0.0001
BLIMP1 (MFI)
HD
ATL
H
HD
ATL
Normalized to Mode
PTEN
Tbet
THEMIS
I
P = 0.0079
P < 0.0001
P < 0.0001
PTEN (MFI)
Tbet (MFI)
THEMIS (MFI)
HD
ATL
Treg cells
J
HD–1
HD–2
HD–3
ATL–1
ATL–2
ATL–3
ATL–4
ATL–5
UMAP 2
UMAP 1
0
1
2
3
4
5
6
K
value
Var1
Var2
HD–1
HD–2
HD–3
ATL–1
ATL–2
ATL–3
ATL–4
ATL–5
L
Treg cells
Cluster
Average Expression
max gene expression
min gene expression
KIAA1841
ABCC5
ENDOD1
CDKN2AIPNL
THEMIS
NFIC
TMEM44-AS1
MN1
ICAM1
LHFPL6
SDC4
SPATA48
IL5RA
ZNHIT2
RADX
KLF4
DYRK1B
GBP5
ADTRP
ANXA1
GSTP1
CD7
SCN9A
RAB37
TSHR
MGST1
NCR3
RIMS2
MARK1
SHROOM3
DGKI
SPRY1

Supplementary Fig. S7: Analysis of Treg cells from acute ATL patients and healthy donors. (**A** to **I**) Purified $CD4^+$ T cells from frozen PBMCs of HD and acute ATL patients were *ex vivo* stimulated with anti-CD3 and anti-CD28 in the presence of IL-2 for 24 hours at 37°C and stained for flow cytometry. (A to C) Gating strategy for $CD3^+CD4^+CD25^+CCR4^+$ T cells in HD and ATL with FMO controls. (D) Frequency of $CD25^+CCR4^+$ T cells in HD and ATL. (E) Flow cytometry analysis of IL-10 production in ATL. (F and G) Representative histograms with statistical analysis showing BLIMP-1 staining at 48 and 72 hours of *ex vivo* culture. (H and I) Intracellular staining for PTEN, Tbet and THEMIS by flow cytometry with statistics. Data are representative of mean ± SEM from three independent experiments (n = 5 individuals per group). Two-tailed unpaired Student's t test was used for statistical analysis. (**J** to **L**) Purified $CD4^+$ T cells from frozen PBMCs of HD and acute ATL patients were *ex vivo* stimulated with anti-CD3 and anti-CD28 in the presence of IL-2 for 4 hours at 37°C and subjected to scRNA-seq. (J) UMAP plot showing different clusters in Tregs, showing three samples from HD and five samples from ATL. (K) Bar plot showing the proportion of different sub-clusters of Tregs in each of the samples from HD and ATL. (L) Dot plot showing the differential gene expression profile in sub-population of Treg cells in HD and ATL patients.

Supplementary Table S1: List of the genes with BLIMP1, IRF, ETS, and RUNX motifs corresponding to BLIMP1 peaks in human natural Tregs (as a separate excel file).

Supplementary Table S2: List of the markers used by Azimuth to define the population clusters within $CD3^{+}CD4^{+}$ T cells in HD and ATL patients (as a separate excel file).

Supplementary Table S3: List of the differentially expressed genes in *PRDM1*$^{-}$ vs *PRDM1*$^{+}$ populations in ATL samples (as a separate excel file).

Supplementary Table S4: List of the genes used by the Qiagen Ingenuity Pathway Analysis (IPA) to identify the pathways in *PRDM1*$^{-}$ vs *PRDM1*$^{+}$ populations in ATL samples (as a separate excel file).

Supplementary Table S5: ATL patient's information.

Patient ID	AGE	SEX	Ethnicity	Type of ATL
1	52 years	Male	African American	Acute
2	76 years	Female	Jamaican	Acute
3	45 years	Male	Unknown	Acute
4	76 years	Female	Caribbean	Acute
5	49 years	Female	Afro Caribbean	Acute

Supplementary Table S6: List of antibodies, reagents and resources.

REAGENT or RESOURCE	SOURCE	IDENTIFIER
**Antibodies**		
*InVivo*MAb anti-mouse CD3ε (145-2C11)	BioXCell	Cat# BE0001-1; RRID: AB_1107634
*InVivo*MAb anti-mouse CD28 (37.51)	BioXCell	Cat# BE0015-1; RRID: AB_1107624
*InVivo*MAb anti-mouse CD3 (OKT-3)	BioXCell	Cat# BE0001-2; RRID: AB_1107632
*InVivo*MAb anti-mouse CD28 (9.3)	BioXCell	Cat# BE0248; RRID: AB_2687729
PerCP/Cy5.5 anti-mouse CD4 (RM4-5)	Biolegend	Cat# 100540; RRID: AB_893326
Brilliant Violet 510™ anti-mouse CD4 (RM4-5)	Biolegend	Cat# 100559; RRID: AB_2562608
PE anti-mouse CD25 (PC-61)	Biolegend	Cat# 102008; RRID: AB_312857
Brilliant Violet 510™ anti-mouse CD25 (PC-61)	Biolegend	Cat# 102042; RRID: AB_2562270
PE anti-mouse CD122 (TM-β1)	Biolegend	Cat# 123210; RRID: AB_940617
PE/Cyanine7 anti-mouse CD122 (TM-β1)	Biolegend	Cat# 123216; RRID: AB_2562895
APC anti-mouse CD122 (TM-β1)	Biolegend	Cat# 123214; RRID: AB_2562575
PE anti-mouse CD19 (6D5)	Biolegend	Cat# 115508; RRID: AB_313643
anti-mouse CD45RB (C363-16A)	Biolegend	Cat# 103320; RRID: AB_2565229
FITC anti-mouse CD45 (30-F11)	Biolegend	Cat# 103108; RRID: AB_312973
Brilliant Violet 605™ anti-mouse CD45.1 (A20)	Biolegend	Cat# 110738; RRID: AB_2562565
Brilliant Violet 421™ anti-mouse CD45.2 (104)	Biolegend	Cat# 109832; RRID: AB_2565511
Brilliant Violet 785™ anti-mouse PD-1 (29F.1A12)	Biolegend	Cat# 135225; RRID: AB_2563680
Brilliant Violet 650™ anti-mouse CXCR5 (L138D7)	Biolegend	Cat# 145517; RRID: AB_2562453
Alexa Fluor® 700 anti-mouse FOXP3 (MF-14)	Biolegend	Cat# 126422; RRID: AB_2750493
APC anti-mouse Blimp1 (5E7)	Biolegend	Cat# 150008; RRID: AB_2728187
Brilliant Violet 421™ anti-mouse IL-2 (JES6-5H4)	Biolegend	Cat# 503826; RRID: AB_ 2650897
Brilliant Violet 605™ anti-mouse IL-2 (JES6-5H4)	Biolegend	Cat# 503829; RRID: AB_11204084
PE/Cyanine7 anti-mouse IL-10 (JESS-16E3)	Biolegend	Cat# 505026; RRID: AB_11150582
PE anti-mouse IL-10 (JESS-16E3)	Biolegend	Cat# 505008; RRID: AB_315362
APC anti-mouse IL-10 (JESS-16E3)	Biolegend	Cat# 505010; RRID: AB_315364
Brilliant Violet 650™ anti-mouse IFNγ (XMG1.2)	Biolegend	Cat# 505832; RRID: AB_2734492
Brilliant Violet 785™ anti-T-bet (4B10)	Biolegend	Cat# 644835; RRID: AB_2721566
Brilliant Violet 605™ anti-T-bet (4B10)	Biolegend	Cat# 644817; RRID: AB_11219388
PE/Dazzle™ 594 anti-mouse/human Bcl-6 (7D1)	Biolegend	Cat# 358510; RRID: AB_2566194
PE/Cyanine7 anti-mouse/human Helios (22F6)	Biolegend	Cat# 137236; RRID: AB_2565990
PE anti-mouse/rat/human FOXP3 (150D)	Biolegend	Cat# 320008; RRID: AB_492980
PE mouse Anti-Stat5 (pY694)	BD Biosciences	Cat# 612567; RRID: AB_399858
PE-Cy™7 mouse Anti-Stat5 (pY694)	BD Biosciences	Cat# 560117; RRID: AB_1645546
PerCP-Cy™5.5 mouse Anti-Stat5 (pY694)	BD Biosciences	Cat# 560118; RRID: AB_1645551
Alexa Fluor® 647 mouse Anti-Akt (pS473)	BD Biosciences	Cat# 560343; RRID: AB_10896328
Brilliant Violet 421™ anti-ERK1/2 Phospho (Thr202/Tyr204)	BD Biosciences	Cat# 369510; RRID: AB_ 2629709
Alexa Fluor® 700 anti-human CD3 (OKT3)	Biolegend	Cat# 317340; RRID: AB_2563408
Brilliant Violet 510™ anti-human CD4 (OKT4)	Biolegend	Cat# 317444; RRID: AB_2561866
PerCP/Cyanine5.5 anti-human CD4 (OKT4)	Biolegend	Cat# 317428 RRID: AB_1186122
Pacific Blue anti-human CD25 (BC96)	Biolegend	Cat# 302627; RRID: AB_2233706

Brilliant Violet 785™ anti-human CD25 (BC96)	Biolegend	Cat# 302638; RRID: AB_2563808
PE/Dazzle™ 594 anti-human CCR4 (L291H4)	Biolegend	Cat# 359420; RRID: AB_2564095
PE anti-human CD122 (TU27)	Biolegend	Cat# 339006 RRID: AB_2248892
PE/Cyanine7 anti-human CD122 (TU27)	Biolegend	Cat# 339014 RRID: AB_2562597
Brilliant Violet 421™ anti-human CD122 (TU27)	Biolegend	Cat# 339010 RRID: AB_2561835
Alexa Fluor® 647 anti-mouse FOXP3 (150D)	Biolegend	Cat# 320014; RRID: AB_439750
PE rat Anti-Blimp-1 (6D3)	BD Biosciences	Cat# 564702; RRID: AB_2738901
Alexa Fluor® 647 rat Anti-Blimp-1 (6D3)	BD Biosciences	Cat# 565002; RRID: AB_2739040
PerCP/Cyanine5.5 anti-mouse/human Helios (22F6)	Biolegend	Cat# 137230; RRID: AB_2561640
PE/Cyanine7 anti-human IL-10 (JES3-9D7)	Biolegend	Cat# 501420; RRID: AB_2125385
Brilliant Violet 650™ Rat Anti-Human IL-10 (JES3-9D7)	BD Biosciences	Cat# 564051; RRID: AB_2738565
Alexa Fluor® 700 anti-human IL-2 (MQ1-17H12)	Biolegend	Cat# 500320; RRID: AB_ 528929
THEMIS, anti-human, PE, REAfinity™ (REA463)	Miltenyi Biotec	Cat# 130-108-244
BD Phosflow™ BV421 Mouse Anti-PTEN (A2B1)	BD Biosciences	Cat# 566636; RRID: AB_ 2869799
Rabbit anti-IgG	Cell Signaling Technology	Cat# 2729S
Blimp-1 Monoclonal Antibody (E.995.1)	ThermoFisher Scientific	Cat# MA5-14879; RRID: AB_11004709
Blimp-1/PRDI-BF1 (C14A4) Rabbit mAb	Cell Signaling Technology	Cat# 9115S
**Bacterial and virus strains**		
Influenza PR8-33 (H1N1)	Mueller et al (*82*)	N/A
**Biological samples**		
Healthy donor blood buffy coats	NIH blood Bank (NIH)	N/A
Frozen PBMCs from acute ATL patients	Lymphoid Malignancies Branch, National Cancer Institute, NIH, USA	N/A
T cells isolated from WT and *Prdm1* CKO mice	NIH	N/A
**Chemicals, peptides, and recombinant proteins**		
Brilliant Violet conjugated NP311-325 tetramer	NIH tetramer core facility	N/A
Recombinant human IL-2 protein	Roche	Cat# Ro 23-6019
LIVE/DEAD Fixable Near-IR	Invitrogen	Cat# L34965
Phorbol 12-myristate 13-acetate (PMA)	Sigma-Aldrich	Cat# P1585
Protein Transport inhibitor	eBioscience	Cat# 00-4980-03
Ionomycin	Sigma-Aldrich	Cat# 13909
Doxycycline	Sigma-Aldrich	Cat# D5207
Electroporation enhancer	Integrated DNA Technologies	Cat# 1075916
Influenza NP (311-325) peptide	AnaSpec, Inc	Cat# AS-62420
Cas9 nuclease	Integrated DNA Technologies	Cat# 1081059
**Critical commercial assays**		
BD cytofix/cytoperm kit	BD biosciences	Cat# 554714
Ebioscience Foxp3/Transcription Factor Staining Buffer Set	eBioscience	Cat# 00-5523-00
Mouse CD4 T cell isolation kit	Stem Cell Technologies	Cat# 19852
Human CD4 T cell isolation kit	Stem Cell Technologies	Cat# 17952
Human natural Tregs isolation kit	Stem Cell Technologies	Cat# 18063
Treg expansion kit, human	Miltenyi Biotec	Cat# 130-095-345
P3 primary Cell 4D-Nucleofector X kit S	Lonza	Cat# V4XP-3032

T7 endonuclease I assay	New England Biolabs	Cat# M0302S
Direct-zol RNA MiniPrep kit	Zymo Research	Cat# R2052
KAPA RNA HyperPrep Kit	KAPABIOSYSTEMS	Cat# KK8542
End-It DNA-Repair kit	Epicentre	Cat# ER0720
Chromium Next GEM Single Cell 3' GEM, Library & Gel Bead Kit v3.1	10X GENOMICS	Cat# PN-1000121
**Deposited data**		
RNA-seq of flu-infected IL-2-treated CD4$^+$ T cells from WT and *Prdm1*$^{fl/fl}$CD4^{cre} mice	This study	GEO: GSE267701
RNA-seq of IL-2-treated Tregs from WT and *Prdm1*$^{fl/fl}$ Foxp3$^{YFP\text{-}cre}$ mice	This study	GEO: GSE267474
BLIMP1 ChIP-seq in IL-2-expanded human Tregs	This study	GEO: GSE267793
scRNA-seq of CD4$^+$ T cells from HD and ATL patients	This study	GEO: GSE267327
**Experimental models: Cell lines**		
ED40515(+) cell line	Phelan et al (*77*)	N/A
**Experimental models: Organisms/strains**		
Mouse: C57BL/6 (B6)	Charles River Laboratory	strain# 027
Mouse: *Prdm1*$^{fl/fl}$	Ciucci et al (*76*)	N/A
Mouse: CD4^{cre}	Jackson laboratory	IMSR_JAX:022071
Mouse: Foxp3$^{YFP\text{-}cre}$	Jackson laboratory	IMSR_JAX:016959
Mouse: B6 CD45.1	Jackson laboratory	IMSR_JAX:002014
Mouse: C57BL/10 *Rag2*$^{-/-}$	Taconic	Line# 103
**Oligonucleotides**		
Alt-R CRISPR-Cas9 tracrRNA	Integrated DNA Technologies	Cat# 10007810
Non-targeting control crRNA 1-4	Integrated DNA Technologies	N/A
PRDM1 crRNA 1-4	Integrated DNA Technologies	N/A
T7 assay forward primers (1-4)	Integrated DNA Technologies	N/A
T7 assay reverse primers (1-4)	Integrated DNA Technologies	N/A
**Recombinant DNA**		
Brunello sgRNA library	Addgene	Cat# 73178
pLKO-based sgRNA vector	Addgene	Cat# 52628
pCMV-VSV-G	Addgene	Cat# 8454
pCMV-dR8.2 dvpr	Addgene	Cat# 8455
pCL-Eco	Addgene	Cat# 12371
BglII	New England Biolabs	Cat# RO144S
pRV.GFP	Ren et al (*80*)	N/A
pLVX-EF1a-IRES-ZsGreen1	Takara	Cat# 631982
**Software and algorithms**		
Adobe® Illustrator 2021	Adobe	https://www.adobe.com/adobe/illustrator
FlowJo 10 software	Treestar	https://www.flowjo.com/
GraphPad Prism 10	Graphpad software	https://www.graphpad.com

Bowtie 2.2.6	Langmead and Salzberg (*108*)	https://bowtie-bio.sourceforge.net/bowtie2/index.shtml
TopHat 2.2.1	Trapnell et al. (*86*)	http://tophat.cbcb.umd.edu
EdgeR/v 3.36.0	Robinson et al. (*87*)	https://bioconductor.org
pheatmap	Kolde et al. (*88*)	https://cran.r-project.org/web/packages/pheatmap/index.html
Limma	Ritchie et al. (*93*)	https://bioconductor.org
fgsea	Korotkevich et al. (*89*)	http://bioconductor.org/packages/fgsea/
GSEA version 3.0	Subramanian et al. (*95*)	https://www.gsea-msgdb.org
cutadapt v1.18	Kechin et al. (*90*)	https://github.com/aakechin/cutPrimers
ClusterProfiler 4.4.4	Wu et al. (*94*)	https://www.bioconductor.org/packages/clusterProfiler/
GO.db 3.15.0	Subramanian et al. (*95*)	https://www.bioconductor.org/packages/ GO.db/
msigdbr 7.5.1	Castanza et al. (*96*)	http://github.com/GSEA-MSigDB
enrichplot package 1.16.2	Yu et al. (*109*)	https://bioconductor.org/packages/enrichplot/
ComplexHeatmap 2.12.1	Gu et al. (*97*)	https://bioconductor.org/packages/ComplexHeatmap/
cLoops2 (v0.0.3)	Cao et al. (*100*)	https://github.com/YaqiangCao/cLoops2
HOMER (v4.10.4)	Heinz et al. (*101*)	http://homer.ucsd.edu/homer/
scDblFinder version 1.16	Germain et al. (*105*)	https://github.com/plger/scDblFinder
10X Genomics Cell Ranger version 7.2.0	Zheng et al. (*103*)	https://support.10xgenomics.com/single-cell-gene-expression/software/pipelines/latest/installation
Seurat version 5.0.1	Hao et al. (*104*)	https://github.com/satijalab/seurat
Azimuth version 0.4.6	Hao et al. (*106*)	https://azimuth.hubmapconsortium.org/
Qiagen Ingenuity Pathway Analysis version 107193442	Kramer et al. (*107*)	https://digitalinsights.qiagen.com/IPA
**Other**		
PBS	Corning	Cat# 21-040-CV
RPMI	Gibco	Cat# 11875093
HBSS	ThermoFisher Scientific	Cat# 14170-112
Collagenase	Sigma-Aldrich	Cat# C7657
DNAse I	Sigma-Aldrich	Cat# DN25
Percoll® Cytvia	Sigma-Aldrich	Cat# GE17-0891-01
FBS	GeminiBio	Cat# 100-106
HEPES	Corning	Cat# 354241
DMEM	ThermoFisher Scientific	Cat# 11320-033
Peniclllin-Streptomycin	Gibco	Cat# 15070-63

Glutamine	Gibco	Cat# 25030-149
2-mercaptoethanol	Gibco	Cat# 21985-023
Lymphocyte separation medium	Corning	Cat# MT25072CI
TexMACS medium	Miltenyi Biotec	Cat# 130-097-197
AB serum	GeminiBio	Cat# 100-812-100
Polybrene	Sigma-Aldrich	Cat# TR-1003
Paraformaldehyde	ThermoFisher Scientific	Cat# 043368-9M
Methanol	ThermoFisher Scientific	Cat# 326950010
Formalin	Sigma-Aldrich	Cat# HT501128

Supplementary Table S7: gRNA sequences for CRISPR/Cas9 deletion of *PRDM1*

crRNA identity No.	crRNA	Strand	gRNA Target Sequence	Exon No.
PRDM1 crRNA 1	PRDM1_106088300	sense	AGGATGCGGATATGACTCTG	2
PRDM1 crRNA 2	PRDM1_106099308	sense	TTTGGACAGATCTATTCCAG	4
PRDM1 crRNA 3	PRDM1_106105127	antisense	GGGGAGCGAGTGATGTACGT	5
PRDM1 crRNA 4	PRDM1_106105284	antisense	GGACGCGTTCAAGTAAGCGT	5
Control crRNA 1	Non-Targeting Control_602		GCCAGGGTATGGGCATCTCG	
Control crRNA 2	Non-Targeting Control_742		GTCAGGTAATAGTCGGACTC	
Control crRNA 3	Non-Targeting Control_749		GTCTTCACAGGGTGCAACGA	
Control crRNA 4	Non-Targeting Control_8		AAACCTAGCCCCAATACTTA	

Supplementary Table S8: Primer sequences for T7 endonuclease I assay.

**Primer Name**	**Primer Sequence**	**Orientation**	**Target gRNA**
T7_F1	ATACGGCTTCTTGGCTCTTTCT	Forward (5'⟶3')	*PRDM1* gRNA 1
T7_R1	GCACCAGGTCCCCAATCTT	Reverse (3'⟶5')	*PRDM1* gRNA 1
T7_F2	AGTGTGCCTTACCTGTTTCC	Forward (5'⟶3')	*PRDM1* gRNA 2
T7_R2	GATGTTCATCCCGTTCTGACAC	Reverse (3'⟶5')	*PRDM1* gRNA 2
T7_F3	CCGTTCTAACATTTCACCCCTC	Forward (5'⟶3')	*PRDM1* gRNA 3
T7_R3	TATTCCCAGGGCTGCTGTGAG	Reverse (3'⟶5')	*PRDM1* gRNA 3
T7_F4	ATTCCATCCTCCACCACTCC	Forward (5'⟶3')	*PRDM1* gRNA 4
T7_R4	AGATTGCTGTAGACAGGGCAC	Reverse (3'⟶5')	*PRDM1* gRNA 4

REFERENCES AND NOTES

1. D. A. Morgan, F. W. Ruscetti, R. Gallo, Selective in vitro growth of T lymphocytes from normal human bone marrows. *Science* **193**, 1007–1008 (1976).

2. H. P. Kim, J. Imbert, W. J. Leonard, Both integrated and differential regulation of components of the IL-2/IL-2 receptor system. *Cytokine Growth Factor Rev.* **17**, 349–366 (2006).

3. W. Liao, J. X. Lin, W. J. Leonard, Interleukin-2 at the crossroads of effector responses, tolerance, and immunotherapy. *Immunity* **38**, 13–25 (2013).

4. S. H. Ross, D. A. Cantrell, Signaling and function of interleukin-2 in T lymphocytes. *Annu. Rev. Immunol.* **36**, 411–433 (2018).

5. T. Taniguchi, H. Matsui, T. Fujita, C. Takaoka, N. Kashima, R. Yoshimoto, J. Hamuro, Structure and expression of a cloned cDNA for human interleukin-2. *Nature* **302**, 305–310 (1983).

6. R. J. Robb, A. Munck, K. A. Smith, T cell growth factor receptors. Quantitation, specificity, and biological relevance. *J. Exp. Med.* **154**, 1455–1474 (1981).

7. M. Sharon, R. D. Klausner, B. R. Cullen, R. Chizzonite, W. J. Leonard, Novel interleukin-2 receptor subunit detected by cross-linking under high-affinity conditions. *Science* **234**, 859–863 (1986).

8. T. Takeshita, H. Asao, K. Ohtani, N. Ishii, S. Kumaki, N. Tanaka, H. Munakata, M. Nakamura, K. Sugamura, Cloning of the γ chain of the human IL-2 receptor. *Science* **257**, 379–382 (1992).

9. M. Noguchi, H. Yi, H. M. Rosenblatt, A. H. Filipovich, S. Adelstein, W. S. Modi, O. W. McBride, W. J. Leonard, Interleukin-2 receptor gamma chain mutation results in X-linked severe combined immunodeficiency in humans. *Cell* **73**, 147–157 (1993).

10. W. J. Leonard, J. X. Lin, J. J. O'Shea, The γ_c family of cytokines: Basic biology to therapeutic ramifications. *Immunity* **50**, 832–850 (2019).

11. W. J. Leonard, J. M. Depper, G. R. Crabtree, S. Rudikoff, J. Pumphrey, R. J. Robb, M. Kronke, P. B. Svetlik, N. J. Peffer, T. A. Waldmann, W. C. Greene, Molecular cloning and expression of cDNAs for the human interleukin-2 receptor. *Nature* **311**, 626–631 (1984).

12. M. Hatakeyama, M. Tsudo, S. Minamoto, T. Kono, T. Doi, T. Miyata, M. Miyasaka, T. Taniguchi, Interleukin-2 receptor β chain gene: Generation of three receptor forms by cloned human alpha and beta chain cDNA's. *Science* **244**, 551–556 (1989).

13. R. Spolski, P. Li, W. J. Leonard, Biology and regulation of IL-2: From molecular mechanisms to human therapy. *Nat. Rev. Immunol.* **18**, 648–659 (2018).

14. M. C. Friedmann, T. S. Migone, S. M. Russell, W. J. Leonard, Different interleukin 2 receptor β-chain tyrosines couple to at least two signaling pathways and synergistically mediate interleukin 2-induced proliferation. *Proc. Natl. Acad. Sci. U.S.A.* **93**, 2077–2082 (1996).

15. J. X. Lin, W. J. Leonard, The role of Stat5a and Stat5b in signaling by IL-2 family cytokines. *Oncogene* **19**, 2566–2576 (2000).

16. T. R. Malek, I. Castro, Interleukin-2 receptor signaling: At the interface between tolerance and immunity. *Immunity* **33**, 153–165 (2010).

17. H. M. Lockyer, E. Tran, B. H. Nelson, STAT5 is essential for Akt/p70S6 kinase activity during IL-2-induced lymphocyte proliferation. *J. Immunol.* **179**, 5301–5308 (2007).

18. H. Suzuki, T. M. Kundig, C. Furlonger, A. Wakeham, E. Timms, T. Matsuyama, R. Schmits, J. J. Simard, P. S. Ohashi, H. Griesser, T. Taniguchi, C. J. Paige, T. W. Mak, Deregulated T cell activation and autoimmunity in mice lacking interleukin-2 receptor β. *Science* **268**, 1472–1476 (1995).

19. B. Sadlack, J. Lohler, H. Schorle, G. Klebb, H. Haber, E. Sickel, R. J. Noelle, I. Horak, Generalized autoimmune disease in interleukin-2-deficient mice is triggered by an uncontrolled activation and proliferation of $CD4^+$ T cells. *Eur. J. Immunol.* **25**, 3053–3059 (1995).

20. M. Noguchi, Y. Nakamura, S. M. Russell, S. F. Ziegler, M. Tsang, X. Cao, W. J. Leonard, Interleukin-2 receptor γ chain: A functional component of the interleukin-7 receptor. *Science* **262**, 1877–1880 (1993).

21. P. A. Antony, C. M. Paulos, M. Ahmadzadeh, A. Akpinarli, D. C. Palmer, N. Sato, A. Kaiser, C. S. Hinrichs, C. A. Klebanoff, Y. Tagaya, N. P. Restifo, Interleukin-2-dependent mechanisms of tolerance and immunity in vivo. *J. Immunol.* **176**, 5255–5266 (2006).

22. T. R. Malek, A. Yu, V. Vincek, P. Scibelli, L. Kong, CD4 regulatory T cells prevent lethal autoimmunity in IL-2Rβ-deficient mice. Implications for the nonredundant function of IL-2. *Immunity* **17**, 167–178 (2002).

23. D. M. Willerford, J. Chen, J. A. Ferry, L. Davidson, A. Ma, F. W. Alt, Interleukin-2 receptor α chain regulates the size and content of the peripheral lymphoid compartment. *Immunity* **3**, 521–530 (1995).

24. D. Gong, T. R. Malek, Cytokine-dependent Blimp-1 expression in activated T cells inhibits IL-2 production. *J. Immunol.* **178**, 242–252 (2007).

25. K. Placek, S. Gasparian, M. Coffre, S. Maiella, E. Sechet, E. Bianchi, L. Rogge, Integration of distinct intracellular signaling pathways at distal regulatory elements directs T-bet expression in human $CD4^+$ T cells. *J. Immunol.* **183**, 7743–7751 (2009).

26. C. A. Turner Jr., D. H. Mack, M. M. Davis, Blimp-1, a novel zinc finger-containing protein that can drive the maturation of B lymphocytes into immunoglobulin-secreting cells. *Cell* **77**, 297–306 (1994).

27. A. Kallies, E. D. Hawkins, G. T. Belz, D. Metcalf, M. Hommel, L. M. Corcoran, P. D. Hodgkin, S. L. Nutt, Transcriptional repressor Blimp-1 is essential for T cell homeostasis and self-tolerance. *Nat. Immunol.* **7**, 466–474 (2006).

28. G. A. Martins, L. Cimmino, M. Shapiro-Shelef, M. Szabolcs, A. Herron, E. Magnusdottir, K. Calame, Transcriptional repressor Blimp-1 regulates T cell homeostasis and function. *Nat. Immunol.* **7**, 457–465 (2006).

29. E. Cretney, A. Kallies, S. L. Nutt, Differentiation and function of Foxp3[+] effector regulatory T cells. *Trends Immunol.* **34**, 74–80 (2013).

30. G. Martins, K. Calame, Regulation and functions of Blimp-1 in T and B lymphocytes. *Annu. Rev. Immunol.* **26**, 133–169 (2008).

31. S. L. Nutt, K. A. Fairfax, A. Kallies, BLIMP1 guides the fate of effector B and T cells. *Nat. Rev. Immunol.* **7**, 923–927 (2007).

32. S. H. Fu, L. T. Yeh, C. C. Chu, B. L. Yen, H. K. Sytwu, New insights into Blimp-1 in T lymphocytes: A divergent regulator of cell destiny and effector function. *J. Biomed. Sci.* **24**, 49 (2017).

33. B. Santner-Nanan, F. Berberich-Siebelt, Z. Xiao, N. Poser, H. Sennefelder, S. Rauthe, D. S. Vallabhapurapu, I. Berberich, A. Schimpl, H. W. Kreth, R. Nanan, Blimp-1 is expressed in human and mouse T cell subsets and leads to loss of IL-2 production and to defective proliferation. *Signal Transduct.* **6**, 268–279 (2006).

34. H. Shin, S. D. Blackburn, A. M. Intlekofer, C. Kao, J. M. Angelosanto, S. L. Reiner, E. J. Wherry, A role for the transcriptional repressor Blimp-1 in CD8[+] T cell exhaustion during chronic viral infection. *Immunity* **31**, 309–320 (2009).

35. G. A. Martins, L. Cimmino, J. Liao, E. Magnusdottir, K. Calame, Blimp-1 directly represses Il2 and the Il2 activator Fos, attenuating T cell proliferation and survival. *J. Exp. Med.* **205**, 1959–1965 (2008).

36. Y. Xiao, M. Qureischi, L. Dietz, M. Vaeth, S. D. Vallabhapurapu, S. Klein-Hessling, M. Klein, C. Liang, A. Konig, E. Serfling, A. Mottok, T. Bopp, A. Rosenwald, M. Buttmann, I. Berberich, A. Beilhack, F. Berberich-Siebelt, Lack of NFATc1 SUMOylation prevents autoimmunity and alloreactivity. *J. Exp. Med.* **218**, e20181853 (2021).

37. T. Uchiyama, J. Yodoi, K. Sagawa, K. Takatsuki, H. Uchino, Adult T-cell leukemia: Clinical and hematologic features of 16 cases. *Blood* **50**, 481–492 (1977).

38. M. Yamagishi, T. Watanabe, Molecular hallmarks of adult T cell leukemia. *Front. Microbiol.* **3**, 334 (2012).

39. T. A. Waldmann, S. Dubois, Y. Tagaya, Contrasting roles of IL-2 and IL-15 in the life and death of lymphocytes: Implications for immunotherapy. *Immunity* **14**, 105–110 (2001).

40. T. S. Migone, J. X. Lin, A. Cereseto, J. C. Mulloy, J. J. O'Shea, G. Franchini, W. J. Leonard, Constitutively activated Jak-STAT pathway in T cells transformed with HTLV-I. *Science* **269**, 79–81 (1995).

41. S. J. Bensinger, P. T. Walsh, J. Zhang, M. Carroll, R. Parsons, J. C. Rathmell, C. B. Thompson, M. A. Burchill, M. A. Farrar, L. A. Turka, Distinct IL-2 receptor signaling pattern in $CD4^{+}CD25^{+}$ regulatory T cells. *J. Immunol.* **172**, 5287–5296 (2004).

42. P. T. Walsh, J. L. Buckler, J. Zhang, A. E. Gelman, N. M. Dalton, D. K. Taylor, S. J. Bensinger, W. W. Hancock, L. A. Turka, PTEN inhibits IL-2 receptor-mediated expansion of $CD4^{+}$ $CD25^{+}$ Tregs. *J. Clin. Invest.* **116**, 2521–2531 (2006).

43. A. Kallies, A. Xin, G. T. Belz, S. L. Nutt, Blimp-1 transcription factor is required for the differentiation of effector $CD8^{+}$ T cells and memory responses. *Immunity* **31**, 283–295 (2009).

44. A. Papillion, M. D. Powell, D. A. Chisolm, H. Bachus, M. J. Fuller, A. S. Weinmann, A. Villarino, J. J. O'Shea, B. Leon, K. J. Oestreich, A. Ballesteros-Tato, Inhibition of IL-2 responsiveness by IL-6 is required for the generation of GC-T_{FH} cells. *Sci. Immunol.* **4**, eaaw7636 (2019).

45. T. A. Waldmann, W. C. Greene, P. S. Sarin, C. Saxinger, D. W. Blayney, W. A. Blattner, C. K. Goldman, K. Bongiovanni, S. Sharrow, J. M. Depper, W. Leonard, T. Uchiyama, R. C. Gallo, Functional and phenotypic comparison of human T cell leukemia/lymphoma virus positive adult T cell leukemia with human T cell leukemia/lymphoma virus negative Sezary leukemia, and their distinction using anti-Tac. Monoclonal antibody identifying the human receptor for T cell growth factor. *J. Clin. Invest.* **73**, 1711–1718 (1984).

46. S. Chen, N. Ishii, S. Ine, S. Ikeda, T. Fujimura, L. C. Ndhlovu, P. Soroosh, K. Tada, H. Harigae, J. Kameoka, N. Kasai, T. Sasaki, K. Sugamura, Regulatory T cell-like activity of Foxp3^{+} adult T cell leukemia cells. *Int. Immunol.* **18**, 269–277 (2006).

47. Y. Liu, Y. Cong, Y. Niu, Y. Yuan, F. Tan, Q. Lai, Y. Hu, B. Hou, J. Li, C. Lin, H. Zheng, J. Dong, J. Tang, Q. Chen, J. Brzostek, X. Zhang, X. L. Chen, H. R. Wang, N. R. J. Gascoigne, B. Xu, S. H. Lin, G. Fu, Themis is indispensable for IL-2 and IL-15 signaling in T cells. *Sci. Signal.* **15**, eabi9983 (2022).

48. S. A. Rosenberg, IL-2: The first effective immunotherapy for human cancer. *J. Immunol.* **192**, 5451–5458 (2014).

49. J. G. Pol, P. Caudana, J. Paillet, E. Piaggio, G. Kroemer, Effects of interleukin-2 in immunostimulation and immunosuppression. *J. Exp. Med.* **217**, e20191247 (2020).

50. S. A. Rosenberg, J. J. Mule, P. J. Spiess, C. M. Reichert, S. L. Schwarz, Regression of established pulmonary metastases and subcutaneous tumor mediated by the systemic administration of high-dose recombinant interleukin 2. *J. Exp. Med.* **161**, 1169–1188 (1985).

51. R. A. Saxton, C. R. Glassman, K. C. Garcia, Emerging principles of cytokine pharmacology and therapeutics. *Nat. Rev. Drug Discov.* **22**, 21–37 (2023).

52. W. J. Leonard, J. X. Lin, Strategies to therapeutically modulate cytokine action. *Nat. Rev. Drug Discov.* **22**, 827–854 (2023).

53. A. N. Shouse, K. M. LaPorte, T. R. Malek, Interleukin-2 signaling in the regulation of T cell biology in autoimmunity and cancer. *Immunity* **57**, 414–428 (2024).

54. A. G. A. Kolios, G. C. Tsokos, D. Klatzmann, Interleukin-2 and regulatory T cells in rheumatic diseases. *Nat. Rev. Rheumatol.* **17**, 749–766 (2021).

55. M. Long, A. J. Adler, Cutting edge: Paracrine, but not autocrine, IL-2 signaling is sustained during early antiviral CD4 T cell response. *J. Immunol.* **177**, 4257–4261 (2006).

56. D. DiToro, C. J. Winstead, D. Pham, S. Witte, R. Andargachew, J. R. Singer, C. G. Wilson, C. L. Zindl, R. J. Luther, D. J. Silberger, B. T. Weaver, E. M. Kolawole, R. J. Martinez, H. Turner, R. D. Hatton, J. J. Moon, S. S. Way, B. D. Evavold, C. T. Weaver, Differential IL-2 expression defines developmental fates of follicular versus nonfollicular helper T cells. *Science* **361**, eaao2933 (2018).

57. H. M. Shin, V. Kapoor, T. Guan, S. M. Kaech, R. M. Welsh, L. J. Berg, Epigenetic modifications induced by Blimp-1 regulate $CD8^+$ T cell memory progression during acute virus infection. *Immunity* **39**, 661–675 (2013).

58. F. Bedoya, G. S. Cheng, A. Leibow, N. Zakhary, K. Weissler, V. Garcia, M. Aitken, E. Kropf, D. S. Garlick, E. J. Wherry, J. Erikson, A. J. Caton, Viral antigen induces differentiation of $Foxp3^+$ natural regulatory T cells in influenza virus-infected mice. *J. Immunol.* **190**, 6115–6125 (2013).

59. H. Zhang, A. Madi, N. Yosef, N. Chihara, A. Awasthi, C. Pot, C. Lambden, A. Srivastava, P. R. Burkett, J. Nyman, E. Christian, Y. Etminan, A. Lee, H. Stroh, J. Xia, K. Karwacz, P. I. Thakore, N. Acharya, A. Schnell, C. Wang, L. Apetoh, O. Rozenblatt-Rosen, A. C. Anderson, A. Regev, V. K. Kuchroo, An IL-27-driven transcriptional network identifies regulators of IL-10 expression across T helper cell subsets. *Cell Rep.* **33**, 108433 (2020).

60. R. Bankoti, C. Ogawa, T. Nguyen, L. Emadi, M. Couse, S. Salehi, X. Fan, D. Dhall, Y. Wang, J. Brown, V. Funari, J. Tang, G. A. Martins, Differential regulation of effector and regulatory T cell function by Blimp1. *Sci. Rep.* **7**, 12078 (2017).

61. E. Cretney, A. Xin, W. Shi, M. Minnich, F. Masson, M. Miasari, G. T. Belz, G. K. Smyth, M. Busslinger, S. L. Nutt, A. Kallies, The transcription factors Blimp-1 and IRF4 jointly control the differentiation and function of effector regulatory T cells. *Nat. Immunol.* **12**, 304–311 (2011).

62. E. Cretney, P. S. Leung, S. Trezise, D. M. Newman, L. C. Rankin, C. E. Teh, T. L. Putoczki, D. H. Gray, G. T. Belz, L. A. Mielke, S. Dias, S. L. Nutt, Characterization of Blimp-1 function in effector regulatory T cells. *J. Autoimmun.* **91**, 73–82 (2018).

63. E. Shen, H. Rabe, L. Luo, L. Wang, Q. Wang, J. Yin, X. Yang, W. Liu, J. M. Sido, H. Nakagawa, L. Ao, H. J. Kim, H. Cantor, J. W. Leavenworth, Control of germinal center localization and lineage stability of follicular regulatory T cells by the Blimp1 transcription factor. *Cell Rep.* **29**, 1848–1861.e6 (2019).

64. P. Kiesler, I. J. Fuss, W. Strober, Experimental models of inflammatory bowel diseases. *Cell. Mol. Gastroenterol. Hepatol.* **1**, 154–170 (2015).

65. F. Powrie, M. W. Leach, S. Mauze, S. Menon, L. B. Caddle, R. L. Coffman, Inhibition of Th1 responses prevents inflammatory bowel disease in scid mice reconstituted with CD45RB^{hi} CD4^{+} T cells. *Immunity* **1**, 553–562 (1994).

66. M. Di Giovangiulio, A. Rizzo, E. Franze, F. Caprioli, F. Facciotti, S. Onali, A. Favale, C. Stolfi, H. J. Fehling, G. Monteleone, M. C. Fantini, Tbet expression in regulatory T cells is required to initiate Th1-mediated colitis. *Front. Immunol.* **10**, 2158 (2019).

67. W. Liao, J. X. Lin, L. Wang, P. Li, W. J. Leonard, Modulation of cytokine receptors by IL-2 broadly regulates differentiation into helper T cell lineages. *Nat. Immunol.* **12**, 551–559 (2011).

68. M. E. Joosse, F. Charbit-Henrion, R. Boisgard, R. H. C. Raatgeep, D. J. Lindenbergh-Kortleve, L. M. M. Costes, S. Nugteren, N. Guegan, M. Parlato, S. Veenbergen, V. Malan, J. K. Nowak, I. Hollink, M. L. Mearin, J. C. Escher, N. Cerf-Bensussan, J. N. Samsom, Duplication of the IL2RA locus causes excessive IL-2 signaling and may predispose to very early onset colitis. *Mucosal Immunol.* **14**, 1172–1182 (2021).

69. R. Martin, Anti-CD25 (daclizumab) monoclonal antibody therapy in relapsing-remitting multiple sclerosis. *Clin. Immunol.* **142**, 9–14 (2012).

70. D. Liang, A. Zuo, H. Shao, W. K. Born, R. L. O'Brien, H. J. Kaplan, D. Sun, Role of CD25^{+} dendritic cells in the generation of Th17 autoreactive T cells in autoimmune experimental uveitis (EAU). *J. Immunol.* **188**, 5785–5791 (2012).

71. S. M. Russell, J. A. Johnston, M. Noguchi, M. Kawamura, C. M. Bacon, M. Friedmann, M. Berg, D. W. McVicar, B. A. Witthuhn, O. Silvennoinen, A. S. Goldman, F. C. Schmalstieg, J. N. Ihle, J. J. O'Shea, W. J. Leonard, Interaction of IL-2R β and γ c chains with Jak1 and Jak3: Implications for XSCID and XCID. *Science* **266**, 1042–1045 (1994).

72. A. Kontzias, A. Kotlyar, A. Laurence, P. Changelian, J. J. O'Shea, Jakinibs: A new class of kinase inhibitors in cancer and autoimmune disease. *Curr. Opin. Pharmacol.* **12**, 464–470 (2012).

73. M. Zhang, L. A. Mathews Griner, W. Ju, D. Y. Duveau, R. Guha, M. N. Petrus, B. Wen, M. Maeda, P. Shinn, M. Ferrer, K. D. Conlon, R. N. Bamford, J. J. O'Shea, C. J. Thomas, T. A. Waldmann, Selective targeting of JAK/STAT signaling is potentiated by Bcl-xL blockade in IL-2-dependent adult T-cell leukemia. *Proc. Natl. Acad. Sci. U.S.A.* **112**, 12480–12485 (2015).

74. V. Valatas, J. He, A. Rivollier, G. Kolios, K. Kitamura, B. L. Kelsall, Host-dependent control of early regulatory and effector T-cell differentiation underlies the genetic susceptibility of RAG2-deficient mouse strains to transfer colitis. *Mucosal Immunol.* **6**, 601–611 (2013).

75. M. Shapiro-Shelef, K. I. Lin, L. J. McHeyzer-Williams, J. Liao, M. G. McHeyzer-Williams, K. Calame, Blimp-1 is required for the formation of immunoglobulin secreting plasma cells and pre-plasma memory B cells. *Immunity* **19**, 607–620 (2003).

76. T. Ciucci, M. S. Vacchio, T. Chen, J. Nie, L. B. Chopp, D. B. McGavern, M. C. Kelly, R. Bosselut, Dependence on Bcl6 and Blimp1 drive distinct differentiation of murine memory and follicular helper $CD4^{+}$ T cells. *J. Exp. Med.* **219**, e20202343 (2022).

77. J. D. Phelan, R. M. Young, D. E. Webster, S. Roulland, G. W. Wright, M. Kasbekar, A. L. Shaffer III, M. Ceribelli, J. Q. Wang, R. Schmitz, M. Nakagawa, E. Bachy, D. W. Huang, Y. Ji, L. Chen, Y. Yang, H. Zhao, X. Yu, W. Xu, M. M. Palisoc, R. R. Valadez, T. Davies-Hill, W. H. Wilson, W. C. Chan, E. S. Jaffe, R. D. Gascoyne, E. Campo, A. Rosenwald, G. Ott, J. Delabie, L. M. Rimsza, F. J. Rodriguez, F. Estephan, M. Holdhoff, M. J. Kruhlak, S. M. Hewitt, C. J. Thomas, S. Pittaluga, T. Oellerich, L. M. Staudt, A multiprotein supercomplex controlling oncogenic signalling in lymphoma. *Nature* **560**, 387–391 (2018).

78. J. G. Doench, N. Fusi, M. Sullender, M. Hegde, E. W. Vaimberg, K. F. Donovan, I. Smith, Z. Tothova, C. Wilen, R. Orchard, H. W. Virgin, J. Listgarten, D. E. Root, Optimized sgRNA design to maximize activity and minimize off-target effects of CRISPR-Cas9. *Nat. Biotechnol.* **34**, 184–191 (2016).

79. D. E. Webster, S. Roulland, J. D. Phelan, Protocols for CRISPR-Cas9 screening in lymphoma cell lines. *Methods Mol. Biol.* **1956**, 337–350 (2019).

80. M. Ren, M. Kazemian, M. Zheng, J. He, P. Li, J. Oh, W. Liao, J. Li, J. Rajaseelan, B. L. Kelsall, G. Peltz, W. J. Leonard, Transcription factor p73 regulates Th1 differentiation. *Nat. Commun.* **11**, 1475 (2020).

81. P. Palese, J. L. Schulman, Mapping of the influenza virus genome: Identification of the hemagglutinin and the neuraminidase genes. *Proc. Natl. Acad. Sci. U.S.A.* **73**, 2142–2146 (1976).

82. S. N. Mueller, W. A. Langley, G. Li, A. Garcia-Sastre, R. J. Webby, R. Ahmed, Qualitatively different memory $CD8^+$ T cells are generated after lymphocytic choriomeningitis virus and influenza virus infections. *J. Immunol.* **185**, 2182–2190 (2010).

83. R. Ebina-Shibuya, E. E. West, R. Spolski, P. Li, J. Oh, M. Kazemian, D. Gromer, P. Swanson, N. Du, D. B. McGavern, W. J. Leonard, Thymic stromal lymphopoietin limits primary and recall $CD8^+$ T-cell anti-viral responses. *eLife* **10**, e611912 (2021).

84. C. Asseman, S. Mauze, M. W. Leach, R. L. Coffman, F. Powrie, An essential role for interleukin 10 in the function of regulatory T cells that inhibit intestinal inflammation. *J. Exp. Med.* **190**, 995–1004 (1999).

85. B. Langmead, C. Trapnell, M. Pop, S. L. Salzberg, Ultrafast and memory-efficient alignment of short DNA sequences to the human genome. *Genome Biol.* **10**, R25 (2009).

86. C. Trapnell, L. Pachter, S. L. Salzberg, TopHat: Discovering splice junctions with RNA-Seq. *Bioinformatics* **25**, 1105–1111 (2009).

87. M. D. Robinson, D. J. McCarthy, G. K. Smyth, edgeR: A bioconductor package for differential expression analysis of digital gene expression data. *Bioinformatics* **26**, 139–140 (2010).

88. R. Kolde, M. R. Kolde, Package ‘pheatmap’. *R Package* **1**, 790 (2015).

89. G. Korotkevich, V. Sukhov, N. Budin, B. Shpak, M. Artyomov, A. Sergushichev. Fast gene set enrichment analysis. bioRxiv 060012 [Preprint] (2021). https://doi.org/10.1101/060012.

90. A. Kechin, U. Boyarskikh, A. Kel, M. Filipenko, cutPrimers: A new tool for accurate cutting of primers from reads of targeted next generation sequencing. *J. Comput. Biol.* **24**, 1138–1143 (2017).

91. A. Dobin, C. A. Davis, F. Schlesinger, J. Drenkow, C. Zaleski, S. Jha, P. Batut, M. Chaisson, T. R. Gingeras, STAR: Ultrafast universal RNA-seq aligner. *Bioinformatics* **29**, 15–21 (2013).

92. B. Li, C. N. Dewey, RSEM: Accurate transcript quantification from RNA-Seq data with or without a reference genome. *BMC Bioinformatics* **12**, 323 (2011).

93. M. E. Ritchie, B. Phipson, D. Wu, Y. Hu, C. W. Law, W. Shi, G. K. Smyth, *limma* Powers differential expression analyses for RNA-sequencing and microarray studies. *Nucleic Acids Res.* **43**, e47 (2015).

94. T. Wu, E. Hu, S. Xu, M. Chen, P. Guo, Z. Dai, T. Feng, L. Zhou, W. Tang, L. Zhan, X. Fu, S. Liu, X. Bo, G. Yu, clusterProfiler 4.0: A universal enrichment tool for interpreting omics data. *Innovation (Camb)* **2**, 100141 (2021).

95. A. Subramanian, P. Tamayo, V. K. Mootha, S. Mukherjee, B. L. Ebert, M. A. Gillette, A. Paulovich, S. L. Pomeroy, T. R. Golub, E. S. Lander, J. P. Mesirov, Gene set enrichment analysis: A knowledge-based approach for interpreting genome-wide expression profiles. *Proc. Natl. Acad. Sci. U.S.A.* **102**, 15545–15550 (2005).

96. A. S. Castanza, J. M. Recla, D. Eby, H. Thorvaldsdottir, C. J. Bult, J. P. Mesirov, Extending support for mouse data in the Molecular Signatures Database (MSigDB). *Nat. Methods* **20**, 1619–1620 (2023).

97. Z. Gu, R. Eils, M. Schlesner, Complex heatmaps reveal patterns and correlations in multidimensional genomic data. *Bioinformatics* **32**, 2847–2849 (2016).

98. A. Barski, S. Cuddapah, K. Cui, T. Y. Roh, D. E. Schones, Z. Wang, G. Wei, I. Chepelev, K. Zhao, High-resolution profiling of histone methylations in the human genome. *Cell* **129**, 823–837 (2007).

99. Y. Cao, S. Liu, K. Cui, Q. Tang, K. Zhao, Hi-TrAC detects active sub-TADs and reveals internal organizations of super-enhancers. *Nucleic Acids Res.* **51**, 6172–6189 (2023).

100. Y. Cao, S. Liu, G. Ren, Q. Tang, K. Zhao, cLoops2: A full-stack comprehensive analytical tool for chromatin interactions. *Nucleic Acids Res.* **50**, 57–71 (2022).

101. S. Heinz, C. Benner, N. Spann, E. Bertolino, Y. C. Lin, P. Laslo, J. X. Cheng, C. Murre, H. Singh, C. K. Glass, Simple combinations of lineage-determining transcription factors prime cis-regulatory elements required for macrophage and B cell identities. *Mol. Cell* **38**, 576–589 (2010).

102. K. Cui, Z. Chen, Y. Cao, S. Liu, G. Ren, G. Hu, D. Fang, D. Wei, C. Liu, J. Zhu, C. Wu, K. Zhao, Restraint of IFN-γ expression through a distal silencer CNS-28 for tissue homeostasis. *Immunity* **56**, 944–958.e6 (2023).

103. G. X. Zheng, J. M. Terry, P. Belgrader, P. Ryvkin, Z. W. Bent, R. Wilson, S. B. Ziraldo, T. D. Wheeler, G. P. McDermott, J. Zhu, M. T. Gregory, J. Shuga, L. Montesclaros, J. G. Underwood, D. A. Masquelier, S. Y. Nishimura, M. Schnall-Levin, P. W. Wyatt, C. M. Hindson, R. Bharadwaj, A. Wong, K. D. Ness, L. W. Beppu, H. J. Deeg, C. McFarland, K. R. Loeb, W. J. Valente, N. G. Ericson, E. A. Stevens, J. P. Radich, T. S. Mikkelsen, B. J. Hindson, J. H. Bielas, Massively parallel digital transcriptional profiling of single cells. *Nat. Commun.* **8**, 14049 (2017).

104. Y. Hao, T. Stuart, M. H. Kowalski, S. Choudhary, P. Hoffman, A. Hartman, A. Srivastava, G. Molla, S. Madad, C. Fernandez-Granda, R. Satija, Dictionary learning for integrative, multimodal and scalable single-cell analysis. *Nat. Biotechnol.* **42**, 293–304 (2024).

105. P. L. Germain, A. Lun, C. Garcia Meixide, W. Macnair, M. D. Robinson, Doublet identification in single-cell sequencing data using scDblFinder. *F1000Res* **10**, 979 (2021).

106. Y. Hao, S. Hao, E. Andersen-Nissen, W. M. Mauck III, S. Zheng, A. Butler, M. J. Lee, A. J. Wilk, C. Darby, M. Zager, P. Hoffman, M. Stoeckius, E. Papalexi, E. P. Mimitou, J. Jain, A. Srivastava, T. Stuart, L. M. Fleming, B. Yeung, A. J. Rogers, J. M. McElrath, C. A. Blish, R. Gottardo, P. Smibert, R. Satija, Integrated analysis of multimodal single-cell data. *Cell* **184**, 3573–3587.e29 (2021).

107. A. Kramer, J. Green, J. Pollard Jr., S. Tugendreich, Causal analysis approaches in ingenuity pathway analysis. *Bioinformatics* **30**, 523–530 (2014).

108. B. Langmead, S. L. Salzberg, Fast gapped-read alignment with Bowtie 2. *Nat. Methods* **9**, 357–359 (2012).

109. Yu. G, enrichplot: Visualization of Functional Enrichment Result. R package version 1.28.2 (2025); https://bioconductor.org/packages/enrichplot.
